# Plasticity in a bacterial global regulatory switch that drives a shift in antibiotic resistance and virulence

**DOI:** 10.1093/femsmc/xtag044

**Published:** 2026-08-25

**Authors:** Annapaula Correia, Emma J Manners, Benjamin A Evans, Jacob G Malone, Justin O’Grady, Eleni Mavrogiorgou, Sergio Arminu, Manuela Usai, Dixita Naik, Stuart McMillan, Gemma L Kay, Claire Hill, Padhmanand Sudhakar, Emma Meader, Katarzyna Schmidt, Tamas Korcsmaros, Andrew P Desbois, Lisa Crossman, John Wain, Gemma C Langridge

**Affiliations:** Norwich Medical School, University of East Anglia, Norwich, NR4 7UQ, United Kingdom; Norwich Medical School, University of East Anglia, Norwich, NR4 7UQ, United Kingdom; European Molecular Biology Laboratory, European Bioinformatics Institute, Wellcome Genome Campus, Hinxton, CB10 1SD, United Kingdom; Norwich Medical School, University of East Anglia, Norwich, NR4 7UQ, United Kingdom; Centre for Microbial Interactions, Norwich Research Park, Norwich, NR4 7UG, United Kingdom; Department of Molecular Microbiology, John Innes Centre, Norwich Research Park, Norwich, NR4 7UH, United Kingdom; School of Biological Sciences, University of East Anglia, Norwich Research Park, Norwich, NR4 7TJ, United Kingdom; Centre for Microbial Interactions, Norwich Research Park, Norwich, NR4 7UG, United Kingdom; Norwich Medical School, University of East Anglia, Norwich, NR4 7UQ, United Kingdom; Quadram Institute Bioscience, Norwich Research Park, Norwich, NR4 7UQ, United Kingdom; Norwich Medical School, University of East Anglia, Norwich, NR4 7UQ, United Kingdom; Department of Biomedical Science, University of Sassari, Sardinia, 07100, Italy; Department of Biomedical Science, University of Sassari, Sardinia, 07100, Italy; Norwich Medical School, University of East Anglia, Norwich, NR4 7UQ, United Kingdom; Clinical and Experimental Sciences, University of Southampton, Southampton, SO16 6YD, United Kingdom; Institute of Aquaculture, University of Stirling, Stirling, FK9 4LA, United Kingdom; Quadram Institute Bioscience, Norwich Research Park, Norwich, NR4 7UQ, United Kingdom; Norwich Medical School, University of East Anglia, Norwich, NR4 7UQ, United Kingdom; Quadram Institute Bioscience, Norwich Research Park, Norwich, NR4 7UQ, United Kingdom; Quadram Institute Bioscience, Norwich Research Park, Norwich, NR4 7UQ, United Kingdom; Department of Biotechnology, Kumaraguru College of Technology, Coimbatore, Tamil Nadu 641017, India; Microbiology Department, NRP Innovation Centre, Norwich Research Park, Norwich, NR4 7GJ, United Kingdom; Microbiology Department, NRP Innovation Centre, Norwich Research Park, Norwich, NR4 7GJ, United Kingdom; Quadram Institute Bioscience, Norwich Research Park, Norwich, NR4 7UQ, United Kingdom; Earlham Institute, Norwich Research Park, Norwich, NR4 7UZ, United Kingdom; Department of Metabolism, Digestion and Reproduction, Imperial College London, London, W12 0NN, United Kingdom; Institute of Aquaculture, University of Stirling, Stirling, FK9 4LA, United Kingdom; Norwich Medical School, University of East Anglia, Norwich, NR4 7UQ, United Kingdom; School of Biological Sciences, University of East Anglia, Norwich Research Park, Norwich, NR4 7TJ, United Kingdom; Sequence Analysis.co.uk, NRP Innovation Centre, Norwich Research Park, Norwich, NR4 7JG, United Kingdom; Centre for Microbial Interactions, Norwich Research Park, Norwich, NR4 7UG, United Kingdom; Norwich Medical School, University of East Anglia, Norwich, NR4 7UQ, United Kingdom; Quadram Institute Bioscience, Norwich Research Park, Norwich, NR4 7UQ, United Kingdom; Centre for Microbial Interactions, Norwich Research Park, Norwich, NR4 7UG, United Kingdom; Quadram Institute Bioscience, Norwich Research Park, Norwich, NR4 7UQ, United Kingdom; Centre for Microbial Interactions, Norwich Research Park, Norwich, NR4 7UG, United Kingdom

**Keywords:** plasticity, antibiotic resistance, virulence, regulation, evolution, pathogen

## Abstract

Antibiotic resistance and expression of virulence factors impact the outcome of infection by *Pseudomonas aeruginosa*. Pathogenesis is often modelled using the PAO1 reference strain but laboratory lineages vary in the sequence and activity of MexT, a global regulator impacting virulence, biofilm formation, and ciprofloxacin resistance. We defined the impact of active versus inactive MexT in PAO1 and observed transcriptomic changes affecting the expression of ~900 genes. Phenotyping revealed altered metabolism, antibiotic resistance, and virulence, resulting in striking variation across a ‘single’ model organism. We propose that antibiotic resistance promotes plasticity in *mexT* accounting for variation across lineages. We introduced antibiotic resistance into clinical *P. aeruginosa* isolates and observed mutations in *mexT* when selective pressure was removed, supporting the proposed evolutionary pathway. Overall, we have demonstrated the transcriptomic basis of MexT as a phenotypic switch in PAO1 and implicated antibiotic resistance as a cause of changes in *mexT*. Furthermore, MexS/MexT-regulated efflux is implicated in the antibiotic stress response and virulence, helping identify the mechanisms for rapid phenotypic switching through *mexT* and confirming that PAO1 is unlike most isolates. Improved understanding of the regulatory changes linked to antibiotic resistance is particularly relevant to *P. aeruginosa* where cycles of antibiotic treatment are common.

## Teaser

The bacterial pathogen *Pseudomonas aeruginosa* uses a regulatory switch to change between antibiotic resistant and virulent phenotypes.

## Introduction

Bacterial survival relies on rapid adaptation to changing environments. Rapid gene regulation changes enable phenotypic plasticity, while genetic changes can lead to new phenotypes. Understanding bacterial adaptation to environmental changes, like antibiotic exposure, is vital for drug development and optimizing existing drugs. While specific antibiotic resistance mechanisms are well-studied, less is known about the evolutionary paths and interactions that transform antibiotic-sensitive strains into multidrug-resistant superbugs. *Pseudomonas aeruginosa*, a WHO priority pathogen, is often multidrug-resistant (WHO bacterial priority pathogens list 2024). Its adaptability allows survival in contaminated environments and human tissues of diabetic and cystic fibrosis patients (Crone et al. 2020). Innate and acquired antimicrobial resistance (AMR) in *P. aeruginosa* enhances antibiotic evasion and host infection (Langendonk et al. 2021). Key AMR mechanisms are enhanced extrusion systems (multidrug efflux, Mex) and reduced antibiotic ingress (Livermore 2002, Schweizer 2003). The 12 Resistance-Nodulation-Division (RND) systems, especially MexAB-OprM, provide intrinsic resistance and are constitutively expressed (Li et al. 1995, Piddock 2006). Other RND systems, like MexEF-OprN, are inducible and adaptive. MexEF-OprN aids trimethoprim, chloramphenicol, and fluoroquinolone resistance through efflux (Maseda et al. 2000) and is counter-regulated with OprD, permeable to imipenem and meropenem. Thus, multidrug resistance (MDR) can be linked to efflux and permeability barriers via a single regulatory event (Ochs et al. 1999). MexEF-OprN’s role extends beyond MDR, involving amino acid/peptide uptake through OprD, natural toxin export (Fetar et al. 2011, Fargier et al. 2012, Juarez et al. 2017), and quorum sensing (Köhler et al. 2001, Lamarche and Déziel 2011, Dreier and Ruggerone 2015) influencing host adaptation and bacterial communication.

**Figure 1:**
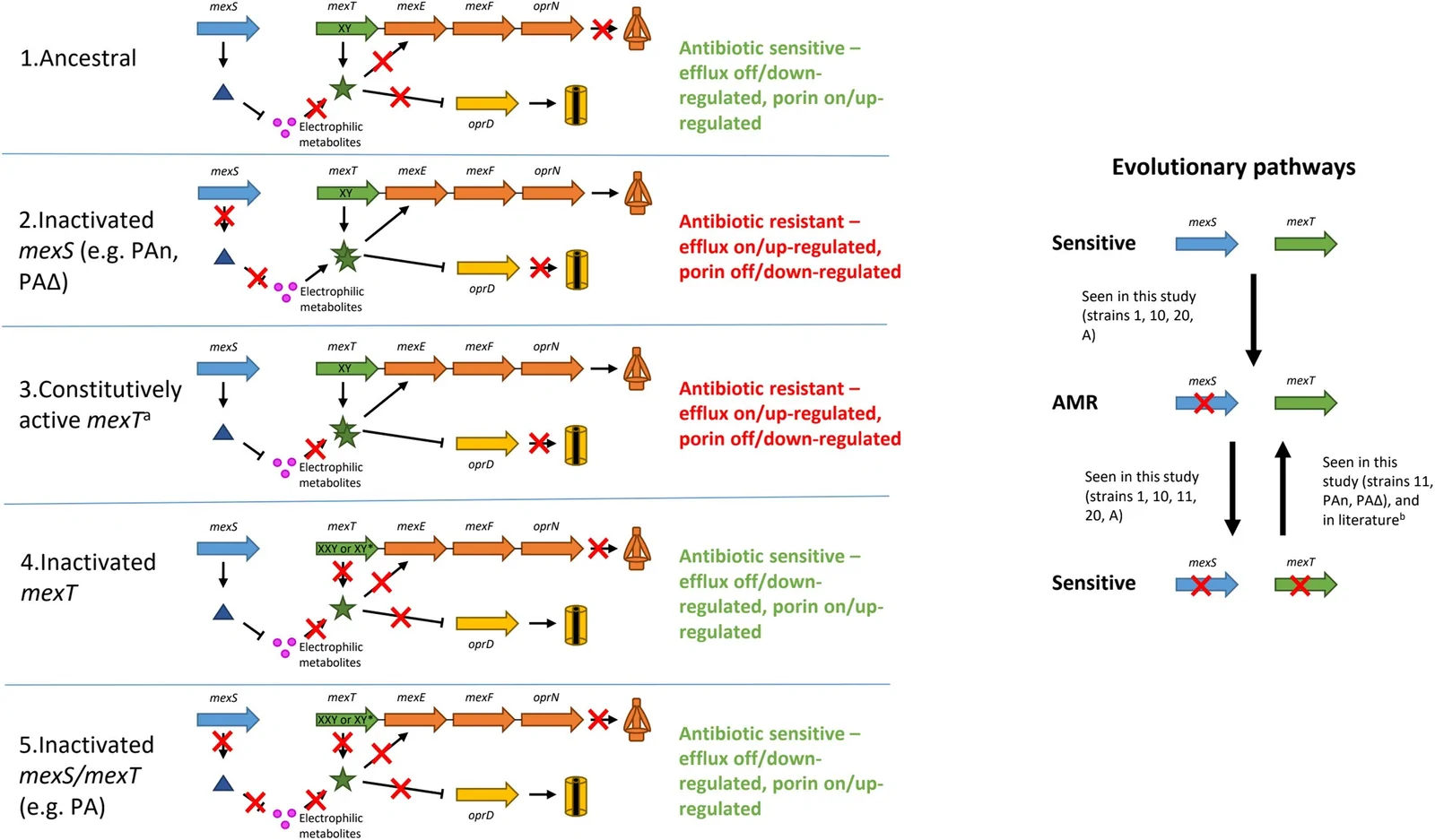
The impacts of mutations in *mexS* and *mexT* on efflux and porin production (left), and the direction of mutational changes observed in isolates in this study or in the literature (right). MexS/*mexS* are blue, MexT/*mexT* are green, MexEF-OprN efflux system genes and proteins are orange, and OprD porin genes and proteins are yellow. ^a^Juarez et al. (2018) proposed that while MexT normally requires a cognate coinducer to bind inactive monomers to form the active oligomer, mutations in *mexT* produce MexT that spontaneously oligomerizes into the active form. ^b^Maseda et al. (2000a).

**Figure 2:**
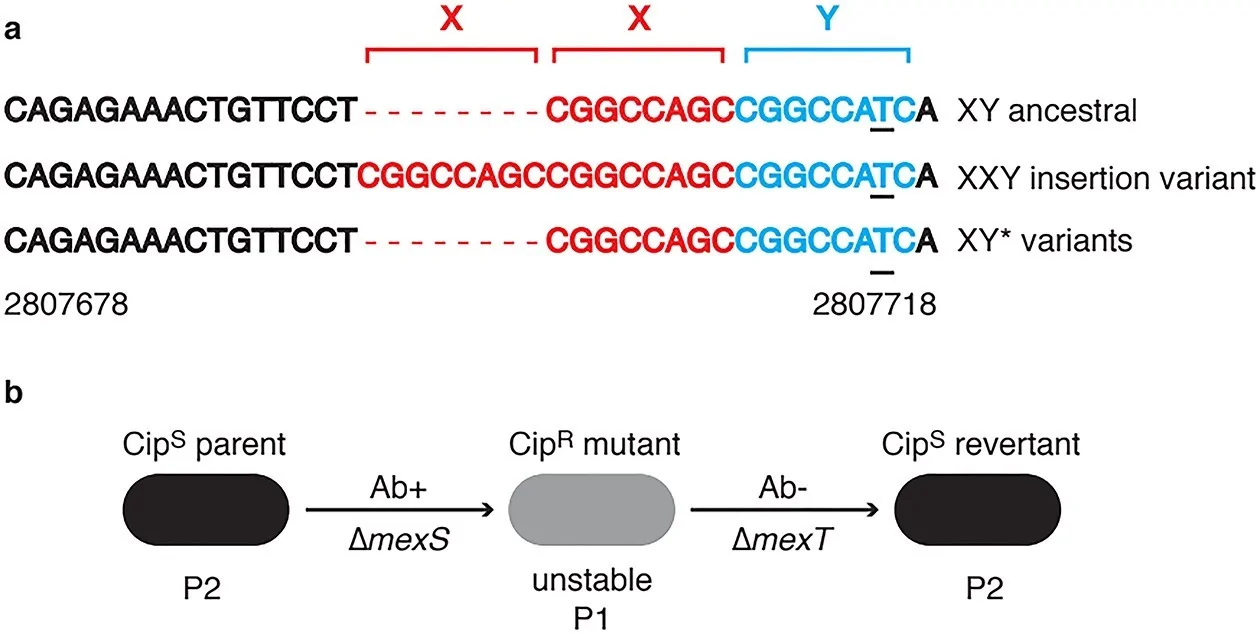
*mexT* sequence variation and evolutionary pathway for antibiotic resistance. (a). The ancestral sequence of *mexT* was found in XY formation. *Pseudomonas aeruginosa* PAO1 *mexT* is disrupted by an 8 bp repeat (XXY). Other mutations (including those outside this region) in *mexT* can also disrupt MexT (XY*). The numbering is according to the reference strain PAO1. (b). Hypothesis for MexT disruption to switch off efflux under antibiotic-free conditions when MexS suppression is lost. Ciprofloxacin resistance (≥ 2 µg/ml) typically emerges through disruption of *mexS* and reversion to ciprofloxacin sensitivity (< 2 µg/ml) emerges through disruption of *mexT*. Ab+ indicates antibiotic presence, Ab− indicates antibiotic absence, and P1 and P2 phenotypes are defined by Luong et al. (2014). The P2 phenotype displays increased virulence.

Understanding gene regulation is key to biological success. The control of MexEF-OprN in *P. aeruginosa*, regulated by MexT and MexS, illustrates the complexity in prokaryotic systems (Fig. 1). Under standard conditions, MexT, a transcriptional activator for MexEF-OprN, is suppressed by MexS activity (Juarez et al. 2017). MexS, an oxidoreductase, detoxifies metabolites and maintains redox balance, keeping MexT inactive and efflux quiescent (Fargier et al. 2012, Juarez et al. 2017). High metabolite concentrations may activate MexT, triggering efflux and restoring redox balance (Fargier et al. 2012, Housseini et al. 2018). Antibiotic resistance can occur through mutations in *mexS* (Sobel et al. 2005, Quick et al. 2014, Richardot et al. 2016) or activating mutations in *mexT* (Juarez et al. 2018), causing constitutive expression of MexEF-OprN (Fig. 1). These mutations disrupt the regulatory system and have costs. The resistant cell has reduced toxin degradation, biofilm formation, and virulence compared to the susceptible phenotype with enhanced virulence (Maseda et al. 2000b, Tian et al. 2009b, Klockgether et al. 2010, Fargier et al. 2012, Luong et al. 2014). Investigation of the sublineages of the common research strain PAO1 revealed mutations in *mexS* and *mexT* that differ from the wider *P. aeruginosa* population (Table 1, Table S2, Fig. S2) (Klockgether et al. 2010, Stover et al. 2000, Luong et al. 2014, Sidorenko et al. 2017, LoVullo and Schweizer 2020). Ancestral *mexT* has two imperfect repeats (‘X’: CGGCCAGC, ‘Y’: CGGCCATC, as XY), but in PAO1-UW, duplication of ‘X’ changes ‘XY’ to ‘XXY’ (Fig. 2a, Fig. S3). This causes a reversible, inactivating frameshift (Maseda et al. 2000a) initially masked by a mis-assigned start codon (LoVullo and Schweizer 2020). Other inactivating mutations in PAO1 *mexT* (‘XY*-type’ mutations) (Maseda et al. 2000a) block MexEF-OprN-mediated efflux, resulting in an antibiotic-sensitive strain (Table S2). Mutations in MexF of the MexEF-OprN efflux pump (Luong et al. 2014) or other efflux genes (Cai et al. 2022) also occur in PAO1, suggesting adaptive benefits from reduced efflux. The higher frequency of MexT over MexEF mutations indicates additional benefits from pleiotropic changes to global regulation by MexT (Oshri et al. 2018). Given *mexT*’s role in AMR and virulence, we investigated frequent genomic lesions in PAO1 *mexT* and their phenotypic impact. We compared active versus inactive MexT in PAO1 to understand resistance generation and cost. We hypothesized MexT inactivation as a response to prior resistance, selecting resistance in clinical isolates and monitoring *mexT* changes in antibiotic-free media. Conditions enabling reversion to susceptible phenotype were identified, explaining mutations causing genome diversity in PAO1. Finally, we examined mutations in clinical isolates in response to chloramphenicol and explored population heterogeneity to understand adaptation dynamics.

**Table 1:** Expected characteristics of *P. aeruginosa* isolates and mutations present. *Pseudomonas aeruginosa* strains UCBPP-PA14, mPAO1/P1, PAO1 (reference), PA1R, and C7447 m were named according to the *Pseudomonas* Genome Database. UCBPP-PA14 is proposed to represent the native control over efflux in *P. aeruginosa*. Key +: gene is expected to be expressed producing functional gene product, -: gene expression is not expected to lead to a functional gene product; Cip^S^: ciprofloxacin susceptible; Cip^R^: ciprofloxacin resistant, P1/P2: phenotypes proposed by Luong et al. (2014) (P1: slow growing, lower virulence phenotype, and antibiotic resistant P2: fast growing, higher virulence phenotype, and antibiotic sensitive).

Genotype	Expected phenotype		Representative *P. aeruginosa* strain (*mexT* genotype)		
*mexS*	*mexT*	Classification	Efflux	Resistance	
+	+	P2	Off	Cip^S^	UCBPP-PA14 (*mexT* XY)
-	+	P1	On	Cip^R^	mPAO1/P1 (*mexT* XY)
-	-	P2	Off	Cip^S^	PAO1 (Reference) (*mexT* XXY)
+	-	P2	Off	Cip^S^	PA1R/C7447m (*mexT* XXY)

## Methods

### Bacterial strains and culture conditions

Strains listed in Table S1 were initially grown on Columbia agar (Oxoid, UK) and then incubated at 37°C for 16 h at 180 rpm in supplemented M9 medium comprised of 22.2 mM glucose, 2 mM MgSO_4_, 0.1 mM CaCl_2_, 24.4 mM casamino acids, and 1 mM thiamine hydrochloride (Sigma-Aldrich, USA). The strains listed in Table S3 were frozen upon receipt without laboratory passage, except for Strain A purified on *Pseudomonas* Agar (Oxoid) with cetrimide and nalidixic acid, and frozen stocks were prepared after overnight growth in lysogeny broth (LB) broth.

### Generation of PAΔ mutant with 8-bp deletion

DNA was extracted from PA-S (Table S1) as per manufacturer’s instructions using the Roche MagNA pure Compact system (Roche, Switzerland), and *mexT* was polymerase chain reaction (PCR)-amplified using Phusion DNA polymerase and Phusion GC Reaction Buffer (New England Biolabs, USA) combined with dNTPs and primers listed in Supplementary methods (Primer list A, Supplementary methods). PCR conditions: Initial denaturation: 98°C (5 min), followed by 32 cycles of: denaturation 98°C (10 s), annealing 55°C (30 s), elongation 72°C (20 s) then final extension of 72°C (5 min). Plasmid DNA from the suicide vector, pTS, was extracted using the NucleoSpin Plasmid kit (Macherey-Nagel, Germany). Amplified *mexT* DNA was digested with MfeI and BamHI and pTS DNA with EcoRI and BamHI, as per the manufacturer’s instructions (New England Biolabs Ltd, UK). The vector was treated with alkaline phosphatase and ligated with the insert at a ratio of 3:1 (insert to vector) using T4 ligase (New England Biolabs) before incubation overnight at 4°C. The pTSmexT constructs were individually transformed into *Escherichia coli* DH5α by heat shock: pTSmexT and *E. coli* DH5α were mixed on ice, incubated at 42°C for 1 min and placed on ice for 2 min. LB broth was added and cultures incubated at 37°C for 2 h. Cells were then plated onto 10 μg/ml tetracycline agar to confirm the presence of *E. coli* DH5α colonies with the construct and tetracycline resistance marker. Colony PCR (primer list A1 and A2, Supplementary methods) and gel electrophoresis were performed to screen colonies for the required insert. PCR steps included: 95°C for 5 min, 30 cycles of 95°C for 30 s, 55°C for 30 s, 72°C for 2 min, and then 72°C for 10 min. Electrocompetent PA cells were prepared by washing in 300 mM sucrose and transformation with the constructs was performed using a Gene Pulser Electroporation System (Bio-Rad, USA) with the following settings: 200 Ω, 2.5 kV before plating onto sucrose agar to counter select the plasmid. Colonies with the insert were then confirmed by sequencing (Eurofins Scientific, Luxembourg) and thereafter named PA∆ (MexS-/MexT+).

### Generation of PAn (MexS-/MexT+)

To generate PAn (MexS-/MexT+), overnight cultures of PA (MexS-/MexT-) were diluted to 10^6^ CFU/ml and plated onto LB agar (Oxoid) containing 0.25 µg/ml ciprofloxacin. One colony, termed PAn (MexS-/MexT+), was identified by PCR (Primer list B, Supplementary methods) with a single X repeat sequence. PCR conditions included: 95°C for 10 s, 60°C for 30 s (45 cycles), followed by a melt curve analysis. The single X repeat sequence was also confirmed using whole- genome sequencing.

### Motility testing

Swarming, swimming, and twitching phenotypes were tested on LB agar concentrations of 0.3%, 0.5%, and 1%, respectively (Rashid and Kornberg 2000, O’May and Tufenkji 2011). For swarm plates, 5 μl of the inoculum was placed on the agar surface. For swimming tests, 5 μl of inoculum representing 10^8^ CFU/ml was stabbed into the centre of the agar. For twitching, the inoculum was pelleted, and a toothpick was used to inoculate the agar–Petri dish interface. Plates were incubated for 18 h at 37°C before the diameters of the motility zones were measured.

### Antibiotic minimal inhibitory concentration testing

Ciprofloxacin stocks were prepared in 0.1 M HCl, chloramphenicol stocks were prepared in ethanol and imipenem stocks were prepared in MOPs buffer. Minimal inhibitory concentration (MICs) for *P. aeruginosa* strains against imipenem, ciprofloxacin, and chloramphenicol (Sigma, UK) were determined by an agar incorporation method. Isolates were streaked onto Mueller–Hinton agar and incubated overnight at 37°C. A single colony was used to inoculate Mueller–Hinton broth and cultured overnight at 37°C. Overnight cultures were normalized to an OD_600_ of 0.5 MacFarland standard and 1 µl inoculum stamped onto Mueller–Hinton plates containing imipenem (range 0.125–128 µg/ml), ciprofloxacin (range 0.125–128 µg/ml), or chloramphenicol (range 1–2048 µg/ml) alongside an antibiotic-free growth control plate using a multipoint inoculator (Denley, UK). MICs were read as the concentrations that showed no visible growth or haze after overnight incubation at 37°C. Each strain was tested in duplicate. *Pseudomonas aeruginosa* ATCC 27853 and *E. coli* ATCC 25922 were included as control strains.

### Phenotype microarray

Utilization of 626 substrates was tested using phenotype microarray (PM) plates and protocols supplied by BioLog Inc., USA. Briefly, strains were serially cultured on Columbia agar twice with incubated overnight at 37°C. Bacterial colonies were suspended in inoculation fluid-0 and dye A, and 100 µl was aliquoted into each well of PM plates 1–2. Sodium succinate (0.54 g/ml) and ferric citrate (0.049 mg/ml) were added to the inoculating fluid for PM plates 3–8. Plates were incubated at 30°C for 96 h in an OmniLog reader. The signal value (SV) (Homann et al. 2005) for each substrate was calculated, and negative controls were subtracted from the results. Negative values were assigned a value of 0, indicating no growth. To enable fold change (FC) calculations, all results (negative and positive) were adjusted by adding 1. SVs for each substrate were averaged across replicates for each strain, and the FC was calculated between PA (MexS-/MexT-) versus PAΔ (MexS-/MexT+) and PA versus PAn (MexS-/MexT+). A normal distribution was used. Results outside of the 95% confidence interval were subjected to a paired Student’s *t*-test, and those with a *P*-value of < .05 were considered significant.

### Analysing growth characteristics

Strains reverting to a reduced chloramphenicol tolerance were initially cultured overnight in the media the reversion was generated in (BHI for Strain 10; revertants A and B, Strain 10; revertant C and D, SSM9PR for the revertant of Strain A) at 37°C alongside its respective chloramphenicol sensitive parent and chloramphenicol tolerant mutant. The density of the overnight culture was adjusted to OD_600_ 0.08–0.1 and further diluted 1:20 to create an inoculum containing approximately 5 × 10^6^ cfu/ml. 20 µl of the final inoculum was then added to five wells of a 96-well plate containing 180 µl of broth and five wells containing 180 µl of broth supplemented with a concentration of chloramphenicol two doubling dilution below the lowest MIC detected for each parent-mutant-revertant strain set (8 µg/ml for both BHI and SSM9PR). Only the inner wells of the 96-well plate were used to avoid condensation interfering with the readings. The plate was incubated at 37°C with shaking at 200 rpm at the beginning of each cycle on the BMG FLUOstar Omega plate reader. Absorbance readings at a wavelength of 600 nm were taken every 5 min over the course of 24 h. Growthcurver (Sprouffske and Wagner 2016) in R v4.0.0 was used to calculate the area under the experimental curve (*auc*). Mann–Whitney *U* tests were used to compare the means and *P*-values were adjusted for multiple testing by the Benjamini–Hochberg method with Rstatix v0.7.0 (Kassambara 2026). Growth curves were plotted with ggplot2 v3.3.5 (Wickham 2009) using the average OD_600_ at 15 min intervals up to 16.5 h to cover the exponential phase of growth. Error bars are shown as 95% confidence intervals. The experiment was conducted twice with five technical replicates included each time.

### Virulence assays

The relative virulence of the *P. aeruginosa* strains PA (MexS-/MexT-), PAn and PAΔ (both MexS-/MexT+) was assessed in the *Galleria mellonella* model according to a protocol modified from McMillan et al. (2015). Briefly, larvae (UK Waxworms Ltd, UK) in groups of 12 were injected with phosphate-buffered saline (PBS) suspensions containing ca. 10 total CFU. In addition, one control group underwent no manipulation to control for background larval mortality (no manipulation control) while another group (uninfected control) was injected with PBS only to control for the impact of physical trauma. Larvae were kept in Petri dishes in the dark at 37°C for up to 24 h and inspected every 6 h so that percentage survival could be calculated for each group. Larvae were considered dead if they did not move after being stimulated with a sterile inoculation loop. The experiment was repeated in triplicate.

### DNA extraction and sequencing

For PAO1-PA (MexS-/MexT-), PAn and PAΔ (both MexS-/MexT+), per strain, DNA was extracted from 1 ml of overnight supplemented M9 culture using the MagNA pure Bacterial lysis kit with RNAse (Qiagen, Germany) on the MagNA Pure Compact instrument (Roche). 1 ng of DNA was used in a standard Nextera XT library preparation for paired-end sequencing on the MiSeq with v3 chemistry (Illumina, USA). For DNA extraction from chloramphenicol-resistant mutants, revertants and the corresponding parent, DNA was extracted from 1 ml of overnight LB culture (supplemented with 100 µg/ml chloramphenicol for chloramphenicol-resistant mutants) using the GeneJet Genomic DNA Purification (ThermoFisher, UK). 1 ng of DNA was used in a standard Nextera XT library preparation before sequencing on the NextSeq with v2 chemistry (Illumina). Adapter and quality trimming of reads was performed with Trimmomatic (Bolger et al. 2014).

### DNA and protein analysis

To identify single nucleotide variants in PAO1 isolates, DNA sequence reads in fastq format were aligned to the reference genome *P. aeruginosa* PAO1, obtained from National Center for Biotechnology Information (NCBI) GenBank with accession number NC_002516. For non-PAO1 isolates used in the evolution experiments, single nucleotide polymorphism (SNP) calls for mutants and revertants were based on alignment to their parent genomes. Reads were aligned to the reference using Bowtie2 (Langmead and Salzberg 2012) and the alignments were sorted, indexed, and stored in bam file format using samtools (Li et al. 2009). Variants were called using Bayesian inference with FreeBayes v1.3.1 (Garrison and Marth 2012). Variants were also called using samtools variant calling methods. High-confidence changes had a samtools quality score of >200. All other changes were of low confidence, with quality scores of <45, and were excluded. Freebayes confirmed the high-confidence changes. Spurious variants were excluded by calling variants of self-reads against the parent reference, which were intersected using bcftools, and intersecting variants were removed from the mutant and revertant variant calls. A further step of filtering variants with vcfutils varFilter was carried out. SNPEff v4.3 was subsequently run across the filtered vcf files, and the results were aggregated into a table (Cingolani et al. 2012).

The ARIBA tool (Hunt et al. 2017) was used to identify changes in specified genes within chloramphenicol-resistant mutants and chloramphenicol-sensitive revertants with *mexS* and *mexT* (and *cmrA*) from *P. aeruginosa* UCBPP-PA14 (obtained from the Pseudomonas Genome Database; Winsor et al. 2016) as the query genes. As a further check, the protein sequences of MexS and MexT were extracted from each strain using BLAST with UCBPP-PA14 sequences as queries and aligned.

Other analyses of genome sequences obtained from the NCBI and *Pseudomonas* Genome Database were performed using the NCBI BLAST and Clustal Omega (Sievers et al. 2011) tools. In addition, a Panaroo (Tonkin-Hill et al. 2020) analysis was performed with reference strains PAO1, PA14, and LES, together with the mutant and revertant sequences.

### COG mapping

COG annotations were downloaded from the *Pseudomonas* Genome Database (last accessed November 2017) and used to categorize the differentially expressed genes in PAΔ and PAn (both MexS-/MexT+). Some genes have not yet been annotated with COG categories: this included 47 and 132 genes up-and downregulated in PAΔ and 43 and 111 genes up-and downregulated in PAn.

### RNA extraction

Initially, growth curves of all strains were conducted to identify the time point at which they would be in the mid-log phase of growth. This was identified to be at 5 h for all strains. For RNA extraction, five colonies from each strain grown on Columbia agar were used to inoculate 10 ml of LB and incubated at 37°C for 24 h. Cultures were diluted to 1:1000 in fresh M9 and grown for 24 h at 37°C, 180 rpm then diluted 1:100 in fresh M9 and incubated for 5 h to ensure log phase. Cultures were then mixed with RNAprotect Bacterial Reagent and centrifuged according to the manufacturer’s protocol (Qiagen, Netherlands). Bacterial pellets were stored at –80°C. TE buffer (10 mM Tris, 1 mM EDTA, pH 8.0) containing 15 mg/ml lysozyme (Fisher Scientific Ltd, UK) and proteinase K (Roche) were added to cell pellets and incubated for 10 min at ambient temperature. Samples were then processed using the RNeasy mini Kit (Qiagen) according to the manufacturer’s protocol, with the inclusion of on-column DNAse treatment from the RNase free DNase kit (Qiagen). Samples were also treated with DNase using the Turbo DNA-free Kit (Life Technologies Ltd, UK) and washed with the RNeasy mini kit. Three independent samples were processed per strain.

### RNA sequencing

The samples were sequenced by Genomed, Poland. Ribo-depletion was performed using the Ribo-Zero rRNA removal kit (Bacteria) (Epicenter, USA), and libraries were prepared using the NEBNext Ultra RNA Library Preparation Kit. Sequencing was performed using Hi-Seq (Illumina) with paired-end sequencing (2× 100 bp) and V3 chemistry reagents.

### RNA sequencing analysis

Transcriptome reads were aligned to the reference genome (NCBI Genbank with accession number NC_002516) by Genomed using TopHat (Kim et al. 2013) and stored in samtools bam file format. Results were provided by an external company as a table containing raw counts and FPKM values, in addition to raw reads. Raw count data was further analysed using the R program DESeq2 to provide log_2_ FC results and *P*-values. Transcript abundance was quantified using DESeq2 v1.12.3 (Love et al. 2014), on R v3.3.1 and edgeR v3.14.0 (Robinson et al. 2020), and genes with log_2_FC > −1 and < 1 were excluded. Both edgeR and DESeq2 were employed to assess concordance of the differential expression profiles. Results from the two analyses were concordant, and so the results taken forwards are from the DESeq2 analysis. Differential expression was assessed using adjusted *p*-values (*P* ≤ .05, Benjamini–Hochberg corrected). To account for mapping all data to the reference strain [PAO1 reference in the Pseudomonas Genome Database (Winsor et al. 2016), in the XXY formation], *mexT* was split into two regions for this analysis. The first region was from the gene start (position 2 807 469) and included the XXY repeat—the second region continued from this to the end of the gene. Results were ranked according to log_2_ FC differences between PA (MexS-/MexT-) and PA∆ or PA and PAn (both MexS-/MexT+). In order to confirm our transcript mapping approach did not miss any additional transcripts, *de novo* transcriptome assemblies were generated using Trinity (Haas et al. 2013), which provided a further means of checking the data for robustness.

### Transcriptional regulatory network reconstruction

DNA binding sites for *P. aeruginosa* PAO1 transcription factors (TF) were retrieved from the Kyoto Encyclopedia of Genes and Genomes (KEGG). For any given TF, we inferred Position Specific Scoring Matrices (PSSMs) representing conserved binding motifs from the literature-sourced raw binding sites using the *consensus* tool (Thomas-Chollier et al. 2008, 2011). PSSMs were then converted to *transfac* format using the *convert-matrix* tool (Münch et al. 2003, Kiliç et al. 2014). To check if any TFs had an effect on gene expression from binding to intergenic regions, we took 2000 bp upstream from the start codon of every gene in the PAO1 reference genome, with no overlap to the upstream ORFs (operon information was obtained from operonDB; Pertea et al. 2009). The *transfac*-formatted PSSMs were used to scan upstream sequences with the *matrix-scan* tool. Putative hits (i.e. genes or operons harbouring TF binding sites) with a *P*-value < 1E^−05^ were considered significant.

### Selection of spontaneous chloramphenicol-resistant *P. aeruginosa*

Clinical *P. aeruginosa* isolates were streaked onto tryptic soy agar (TSA) and incubated overnight at 37°C. Five colonies were resuspended in 2 ml PBS and 10 µl spotted onto TSA and TSA supplemented with chloramphenicol at 100, 200, or 400 µg/ml before overnight incubation at 37°C. In parallel, 25 µl of un-passaged clinical *P. aeruginosa* stocks were spread directly onto TSA plates supplemented with 400 µg/ml chloramphenicol alongside a TSA growth control and incubated at room temperature for > 48 h. Putative chloramphenicol resistant mutants were streaked on TSA supplemented with chloramphenicol at 400 µg/ml and incubated at 37°C alongside the parent (Table S3). Confirmed resistant mutants were harvested from plates in 0.5 ml LB and supplemented with 25% aq. glycerol for storage at −80°C.

### Selection of antibiotic-sensitive revertants

Chloramphenicol-resistant *P. aeruginosa* were streaked on TSA supplemented with chloramphenicol at 400 µg/ml and approximately five colonies used to inoculate either 1 ml brain–heart infusion (BHI) or SSM9PR (1× M9 salts, 2 mM MgSO_4_, 0.1 mM CaCl_2_, 1% glucose, 1% casamino acids, 1 mM thiamine HCl, and 0.05 mM nicotinic acid). The *P. aeruginosa* PA (MexS-/MexT-), PAn and PAΔ (both MexS-/MexT+) strains were included as growth controls. Cultures were passaged up to six times over 2.5 weeks with incubation at either room temperature (static) or 37°C (180 rpm) in the intervening time. For passage, 10 µl culture was used to inoculate fresh BHI or SSM9PR media in the same vial. At 3, 5, and 6 days, 2 µl culture was replica plated on chloramphenicol (400 µg/ml) and antibiotic-free plates (alongside the parent) and isolates with visibly reduced growth on chloramphenicol were streaked on TSA for single colonies and incubated at 37°C overnight. Single colonies from streaked cultures were replica plated on chloramphenicol (400 µg/ml) and antibiotic-free media to identify susceptible revertants, which were confirmed by MIC testing. Putative revertants were harvested from TSA plates in 0.5 ml LB and supplemented with 25% glycerol and stored at −80°C.

## Results and discussion

### Disruption of MexT is not restricted to PAO1

**Figure 3:**
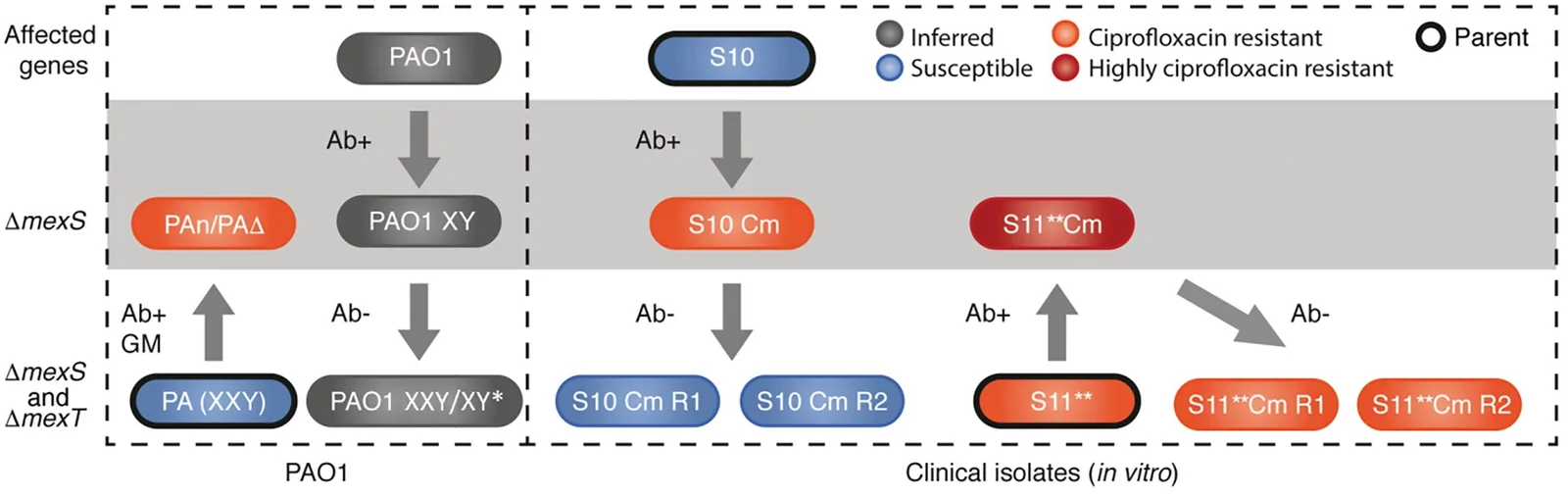
*In vitro* evolution of antibiotic resistance in *P. aeruginosa*. Impact of addition and removal of antibiotic selection pressure on phenotypes and genotypes of *P. aeruginosa* isolates. The dashed line box on the left indicates the responses of PAO1 isolates [including PA∆ (MexS-/MexT+), generated through genetic modification: GM]. The dashed line box on the right indicates the responses of the clinical isolates. Colours denote susceptibility (blue, < 2 µg/ml) and resistance (red, ≥ 2 µg/ml) to ciprofloxacin; high-level resistance to ciprofloxacin (≥ 32 µg/ml) was conferred by mutations in *gyrA* (**), denoted by dark red. The phenotypes of PAO1 isolates are shown in grey. Ab+: chloramphenicol selection; Ab-: antibiotic-free; Cm: chloramphenicol-resistant mutant; and R: chloramphenicol-sensitive revertant.

The *mexT* XXY form (Fig. 3) is described for PAO1 but not exclusive to the reference strain (Table S2). To determine these variants’ prevalence, we examined all NCBI database sequences (last accessed January 2021) and found rare 8-bp insertions (XXY) in *mexT*, including in a mucoid cystic fibrosis C7447 m isolate (Yin et al. 2013) and a phage-resistant derivative of the respiratory isolate PA1 (Le et al. 2014, Lu et al. 2015). *mexT* modification or deletion is reported for lung-adapted clinical isolates (Smith et al. 2006, Warren et al. 2011, Hilliam et al. 2017, La Rosa et al. 2018). Overall, 98% of *P. aeruginosa* were XY for *mexT*, and 91% of *mexF* sequences lacked disruptive mutations, encoding an inducible efflux system. This supported our proposal that the ancestral form of MexT is XY, meaning most studied strains differ from the PAO1-UW reference strain.

### Refunctionalization of MexT in PAO1 produces an antibiotic-resistant phenotype

We hypothesized that MexT disruption switches off efflux in antibiotic-free conditions when MexS suppression is lost during resistance acquisition (Fig. 2b), leading to the antibiotic-sensitive PAO1-UW strain (inactive MexS-N249D and *mexT*-XXY). We assessed the impact of reintroducing functional (XY) MexT into the PAO1 MexS-null background.

Using a PAO1 strain with a MexS N249D null mutation (Richardot et al. 2016) and inactive XXY MexT (strain PA), we created an isogenic mutant (PA∆ (MexS-/MexT+)) by deleting one X repeat (producing an XY genotype) (Figs 2a and 3). PA (MexS-/MexT-) was subjected to ciprofloxacin selection, isolating an *nfxC*-type mutant (PAn (MexS-/MexT+)) with a single *mexT* X repeat (Köhler et al. 2001) (Table S1). Deletion of the second 8 bp repeat in *mexT* restored the reading frame in PA∆ and PAn, increasing *mexT* expression >1.6 log_2_ fold compared to PA (*P* < .001; Dataset S1). Functionalization MexT in PA∆ and PAn (both MexS-/MexT+) caused resistance to chloramphenicol, ciprofloxacin, and imipenem, consistent with MexEF-OprN pump overexpression without functional MexS (Fig. 1; Datasets S1 and S2). We propose that antibiotic-resistant PAO1 with inactive MexS but functional MexT (XY genotype) is the likely ancestor of sensitive PAO1 strains with inactive MexS and MexT, where MexT disruptions occurred via various mechanisms (XY* and XXY) (Figs 1 and 3). Work published after we had performed these experiments (Lee et al. 2021) supports this evolutionary trajectory, restoring ancestral MexS protein in PAO1 and highlighting the atypical PAO1 nature.

### Antibiotic exposure selects for resistance via inactivating MexS

**Figure 4:**
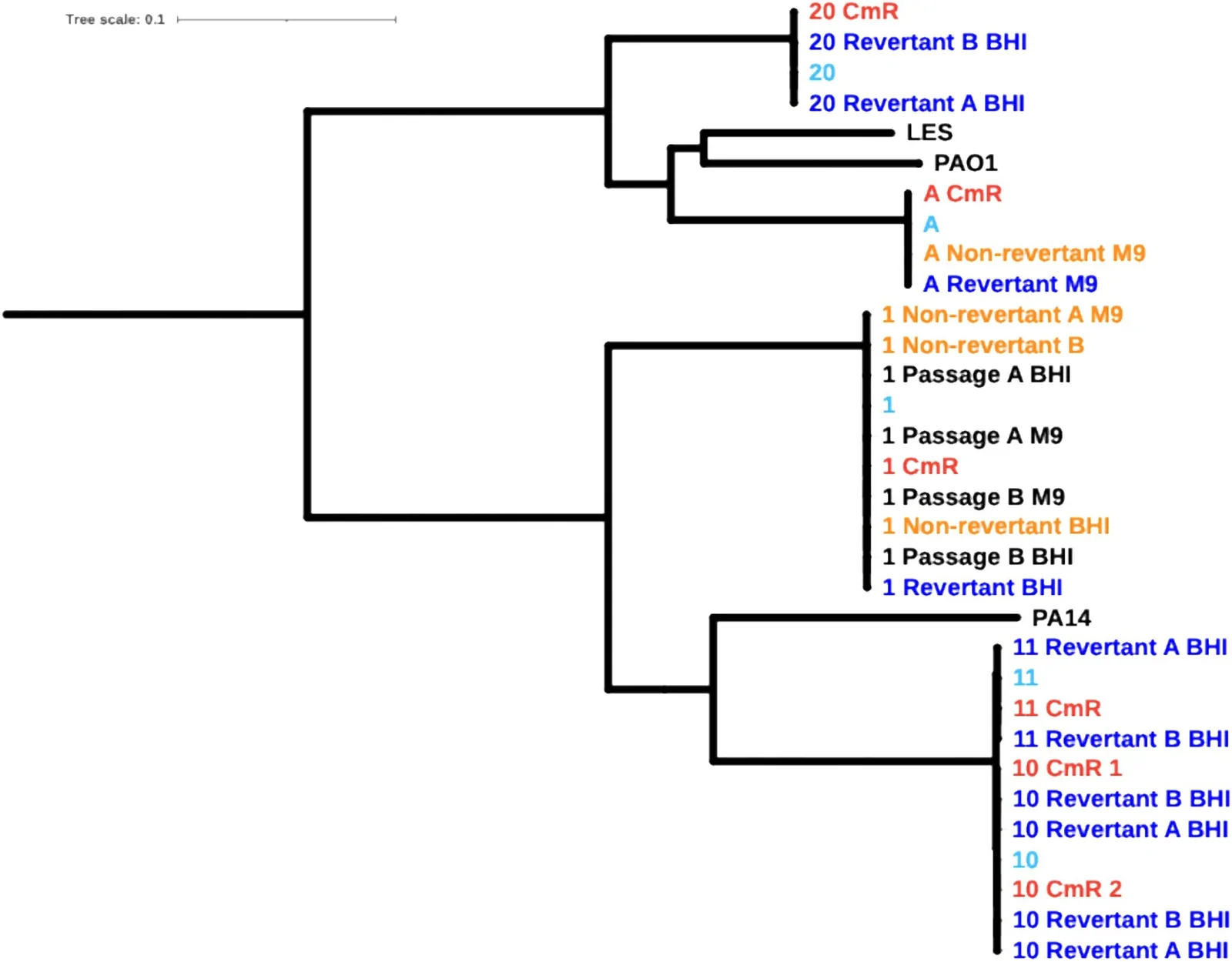
Phylogenetic analysis of *P. aeruginosa* isolates. Core SNP tree of *P. aeruginosa* parent strains (light blue), *in vitro*-generated chloramphenicol-resistant mutants (CmR, red), passaged chloramphenicol-sensitive revertants ('Revertant', dark blue), and resistant non-revertant strains ('Non-revertant', orange). Reference strains, including *P. aeruginosa* PAO1-UW, *P. aeruginosa* UCBPP-PA14, and *P. aeruginosa* LESB58, were also provided for comparison.

In the PAO1 inactive MexS/MexT background, antibiotic resistance arises through refunctionalizing MexT. Most *P. aeruginosa* isolates have the ancestral active MexS/MexT background (Fig. 1), and previous studies show that inactivating MexS results in resistance (Richardot et al. 2016, Lee et al. 2021, Kostylev et al. 2023). To explore MexS and MexT’s roles in resistance across genotypes, clinical strains were exposed to chloramphenicol, and isolates selected that evolved increased resistance (400 µg/ml). We picked 40 mutants from 33 genotypes. Whole genome sequencing of six mutants from five parent strains confirmed chloramphenicol resistance mechanisms and placed these strains within a broader *P. aeruginosa* phylogeny (Fig. 4). Sequencing showed chloramphenicol resistance was linked to *mexS* truncations or frame shifts in 5/6 cases, consistent with the literature (Table 2). In Strain 11, the parent likely had an inactive MexS/MexT background, similar to PAO1, with resistance emerging through MexT refunctionalization. Chloramphenicol selection in Strain 11 resulted in a mutant with a 1-bp deletion in *mexT*, causing a frameshift and active protein. The MexS mutation in the ciprofloxacin-resistant clinical parent Strain 11 (Table 2, Fig. 1) was also seen *in vitro* (A27fs in strain A), confirming *in vitro* resistance relevance to *in vivo* mechanisms. Changes in *cmrA*, linked to MexEF-OprN-mediated resistance, were not detected (Juarez et al. 2017). In all six sequenced mutants, ciprofloxacin MIC values increased alongside chloramphenicol MICs, and in 4/6 mutants, imipenem MICs also rose (Table 2), aligning with MexEF-OprN’s known function in chloramphenicol and ciprofloxacin efflux and imipenem import (Köhler et al. 2001). No new mutations in topoisomerases/gyrases were found, consistent with chloramphenicol selection (Bruchmann et al. 2013). Notably, the two mutants where imipenem MIC did not increase (Strain 10 CmR 1 and Strain 10 CmR 2) had a frameshift mutation in *oprD* in the parent strain, already resistant to imipenem. These data show that in the active MexS/MexT background, *P. aeruginosa* predominantly switches from a sensitive to resistant phenotype through *mexS* mutation, resulting in active MexT, under our experimental conditions.

**Table 2:**
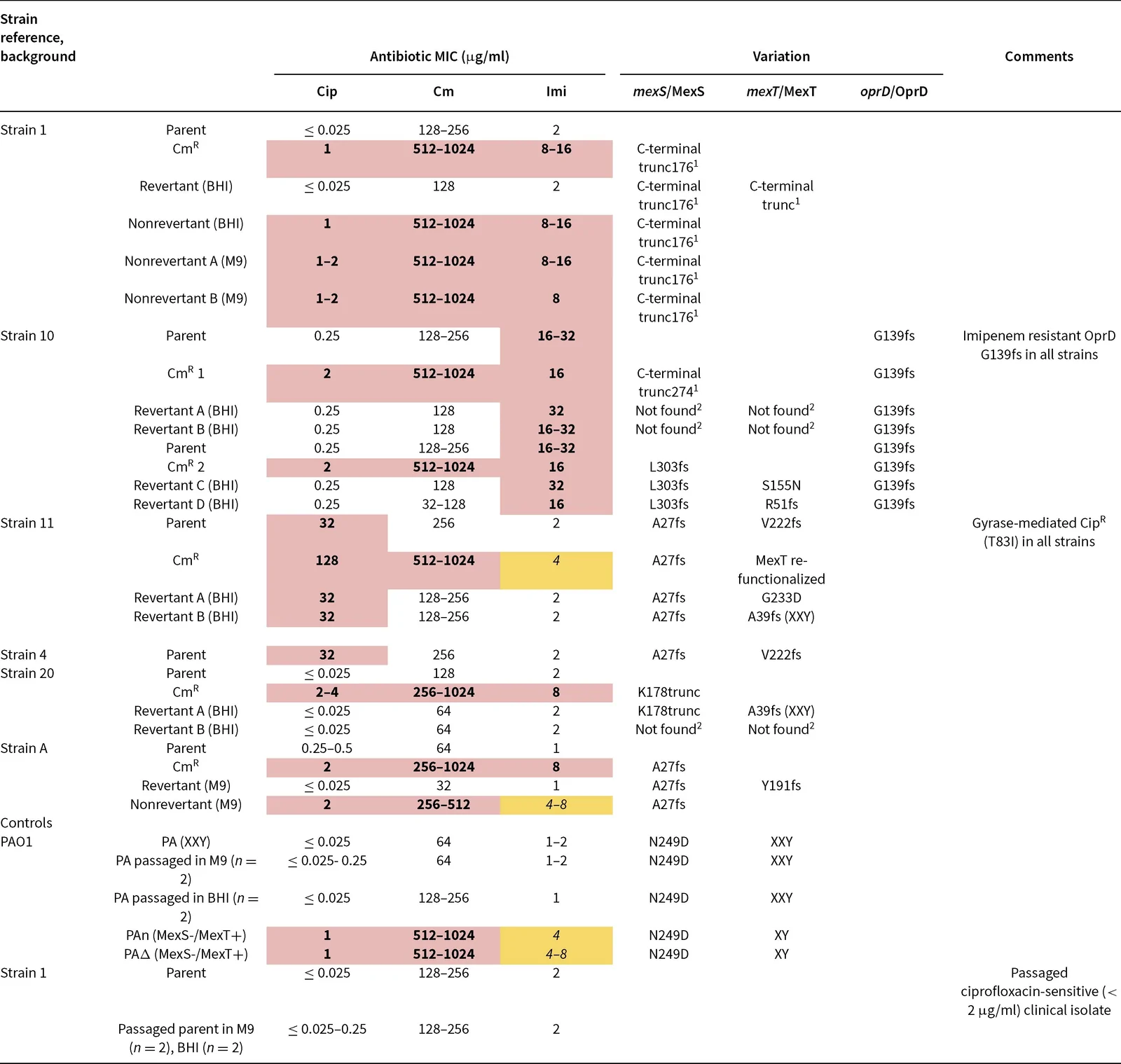
Antibiotic susceptibility and genotype of *P. aeruginosa* strains evolved in this study (MIC range, *n* =2). The genotype was determined by analysis of the raw sequence reads using the ARIBA tool as described in the methods, protein sequences were also extracted by BLAST for confirmation of changes as described in the methods. The clinical breakpoint at which strains are considered to be resistant is 1 µg/ml for ciprofloxacin (cip), and 8 µg/ml for imipenem (imi)^1^. Resistant values are in bold, and intermediate values are in italics. Cm: chloramphenicol, CmR: chloramphenicol resistance; trunc: truncation; fs: frameshift, BHI: passage in BHI broth, M9: passage in SSM9PR broth, XY: ancestral *mexT* with two imperfect ‘XY’ repeats, XXY: disrupted *mexT* with duplication of the first 8 bp repeat, ^1^ results obtained using protein sequences extracted by BLAST, ^2^ sequences not found by ARIBA analysis or extraction by BLAST.

### Global transcriptomic impact of MexT activation

To assess transcriptional impact in antibiotic-sensitive MexT-inactive (XXY) and antibiotic-resistant MexT-active (XY) phenotypes, we conducted transcriptomics on PAΔ and PAn (MexS-/MexT+) and parental PA (MexS-/MexT-) strains (Figs 1 and 3). Compared to PA, 611 genes in PAΔ had reduced expression (log_2_FC < −1, *P* < .05) and 327 increased expression (log_2_FC > 1, *P* < .05), affecting 1/6 of the genome (938/5573 genes), with PAn showing slightly less impact (772/5573). Despite the genetic similarities, only 500 genes were found to be downregulated in both PAΔ and PAn, whilst 245 upregulated genes were found in common. Genetic differences and stochastic effects may explain this, but comparison with PA (MexS-/MexT-) identified common MexT-controlled genes. Translation, ribosomal structure, and biogenesis (category J) had the largest expression increase, highlighting *mexT*’s regulatory role. Over 10% of genes in secretion (U), signal transduction (T), secondary metabolites (Q), and unknown function (S) categories were downregulated, suggesting *mexT*’s indirect repression via other regulators (Datasets S1 and S2), as seen in other LysR regulators (Vercammen et al. 2015).

Transcriptionally active MexT [in PAΔ and PAn (MexS-/MexT+)] strongly impacted *mexEF* and *oprN* expression in both variants (log_2_FC > 7, *P* < .001 for all three genes), consistent with its role regulating the MexEF-OprN pump and hypothetical signal peptides (Datasets S1 and S2) reportedly under the control of MexT (Tian et al. 2009a). In line with the literature, virulence genes for phenazine, rhamnolipid and hydrogen cyanide production were downregulated (Tian et al. 2009b, Köhler et al. 2001) while nitrogen respiratory chain genes were partially upregulated, a known compensation strategy for efflux in *P. aeruginosa* (Olivares et al. 2014). In contrast to a previous report of a *mexT*-controlled *mexEF* over-expresser (Linares et al. 2005), the T3SS encoded by *pscB*-*L* and its activator *exsA* were upregulated. Functional MexT also activated detoxification genes (Fetar et al. 2011) including the nitrosative response (PA3229 and PA4881, log_2_FC >, *P* < .001), *xenB* operon (PA4354-56, log_2_FC ≥ 1.4, *P* < .001), and several ABC transporters (PA2811-2, log_2_FC ≥ 1.9, *P* < .001), supporting a role for MexEF-OprN during host colonization (Fetar et al. 2011). The *mexS* gene (PA2491) was also upregulated, consistent with active MexT and MexEF, and promoting the primary mechanism of detoxification (log_2_FC ∼ 3, *P* < .001) (Fargier et al. 2012).

Toxin export through MexEF-OprN is a proposed ‘back-up’ mechanism for cell survival, but may also serve to divert energy from virulence traits during cellular stress by exporting quorum-sensing molecules (Lamarche and Déziel 2011, Kumar and Schweizer 2011, Kim et al. 2015). Altered efflux has been reported for PAO1 in rat tissue and *in vivo* models and may be either active (Join-Lambert et al. 2001) or inactive (Frisk et al. 2004, Luong et al. 2014) suggesting that niche and temporal responsiveness are key. PAO1 strains that have lost the regulation of efflux may rely on alternative strategies for the control of export, potentially at the expense of systems related to virulence.

### Regulatory network demonstrates a route to altered virulence

**Figure 5:**
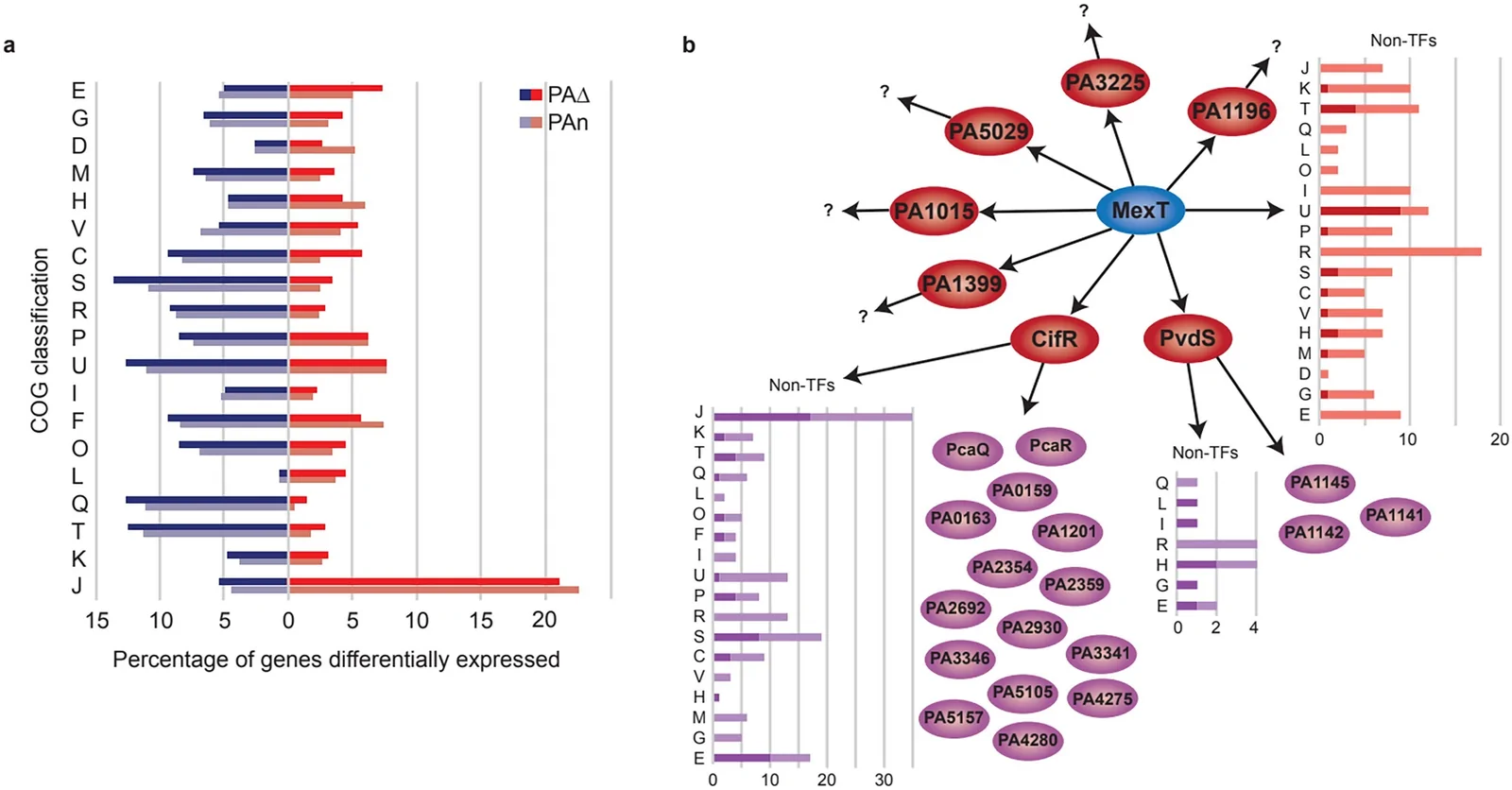
Impact of MexT on gene expression and regulation in PAO1. (a). Differentially expressed genes in PAn and PA∆ (both MexS-/MexT+, active MexT XY) compared to the parent PA (MexS-/MexT-, inactive MexT XXY). Bars indicate the percentage of the total genome for each COG category. Blue (left of 0) = downregulated; red (right of 0) = upregulated. The absolute numbers per category are provided in the Supporting Information. (b). Transcriptional regulatory network of MexT. TFs in red are directly affected by MexT. Downstream effects are shown in purple, where known; TFs in purple and non-TFs in bar charts. The number of non-TFs is displayed by COG category, with bold colour indicating how many genes were found to be differentially expressed in PA∆ or PAn compared to PA. E: amino acid transport and metabolism; G: carbohydrate transport and metabolism; D: cell cycle control, cell division, chromosome partitioning; M: cell wall/membrane/envelope biogenesis; H: coenzyme transport and metabolism; V: defense mechanisms; C: energy production and conversion; S: function unknown; R: general function prediction only; P: inorganic ion transport and metabolism; U: intracellular trafficking, secretion, and vesicular transport; I: lipid transport and metabolism; F: nucleotide transport and metabolism; O: posttranslational modification, protein turnover, chaperones; L: replication, recombination and repair; Q: secondary metabolites biosynthesis, transport and catabolism; T: signal transduction mechanisms; K: transcription; and J: translation, ribosomal structure, and biogenesis.

*Pseudomonas aeruginosa* has three main quorum-sensing systems, *las, rhl* and PQS (*Pseudomonas* quinolone signal, *pqs*), important for acute virulence (Shrout et al. 2006, Letizia et al. 2025). Both *lasR* and *rhlR* were downregulated in antibiotic-resistant mutants, possibly explaining reduced swimming and swarming, indicating lower virulence (Winstanley et al. 2016) (Fig. S1). The transcriptional network downstream of MexT (Fig. 5b) was reconstructed using published MexT binding sites (Tian et al. 2009a, Maseda et al. 2010). MexT binding sites were linked to 166 genes, including 7 TFs (Dataset S3). Two TFs (CifR and PvdS) had known TF binding sites (Wilson et al. 2001, Ochsner et al. 2002, Ballok et al. 2012), which we used to determine indirect regulation. MexT directly and indirectly (mainly through CifR) mediates various functions (COG classification, Fig. 5b). Five MexT-regulated TFs had unknown binding sites, offering future research opportunities into regulatory cascades causing the observed gene expression changes and phenotypes.

Overlaying our RNA-seq data onto this regulatory network showed *mexT* functionalization significantly impacted one of the seven TFs, *pvdS* [log_2_FC ∼0.8 in PA∆/PAn (MexS-/MexT+), *P* < .001], a sigma factor in the *pvd* locus. In PA∆ and PAn, siderophore (pyoverdine) expression via PvdS (e.g. *pvdEFHNPT* Log_2_FC ≥ 1, *P* < .001) (Miyazaki et al. 1995) correlated with reduced phenazine expression. Phenazines generate bioavailable ferrous iron from ferric iron (Wang et al. 2011) and pyoverdines sequester ferric iron directly (Cornelis and Dingemans 2013). Active MexT in PAn and PAΔ reduced ferrous iron metabolism (phenazine biosynthesis and *feoAB*) and increased pyoverdine production (ferric iron uptake) and haem iron acquisition (*phuRT*). Reduced phenazine gene cluster expression in PA∆ and PAn suggests reduced virulence (Panayidou et al. 2020). Active MexT and efflux in PA∆ and PAn may be linked to colonization of clinically relevant sites and expression of iron utilization genes (Cornelis and Dingemans 2013), while inactive MexT is linked to virulence. For example, bioavailable ferrous iron is present in infected lungs (Cornelis and Dingemans 2013) and the pyoverdine iron acquisition route is often lost during chronic infection (Hilliam et al. 2017), while phenazine production is key to virulence (Lau et al. 2004). In a rat intestinal model, inactive MexT frequency was high at surgical trauma sites and low in distant healthy sites (Olivas et al. 2012). Following conclusion of our data collection, we noted Kostylev et al. (2023) drew similar conclusions about mutated MexS in PAO1 affecting MexT and efflux, impacting virulence, especially regarding quorum sensing via RhlI-R.

Some phenazines, such as pyocyanin, are important toxins for *P. aeruginosa* infection and virulence (Lau et al. 2004). We tested the virulence of PA (MexS-/MexT-), PAΔ, and PAn (both MexS-/MexT+) in a *Galleria melonella* model (Fig. S1, D). PAΔ with active MexT was significantly less virulent than PA with inactive MexT (*P* = 3.27e^−7^). PAn (active MexT) was less virulent but not significantly different from PA. Efflux-active mutants (PAΔ and PAn) differed significantly in virulence (*P* = 4.02e^−4^), indicating the role of other factors. Previous data have shown an inverse correlation between virulence factors and *mexE* expression (Richardot et al. 2016). Reduced virulence in MexT-active strains may partly result from phenazine biosynthesis downregulation (Cezairliyan et al. 2013).

### Changes in MexT cause a metabolic shift in substrate utilization

In addition to MexT’s role in regulating genes linked to antibiotic resistance and virulence, we hypothesized that significant changes in global gene expression in strains PAΔ and PAn (both MexS-/MexT+, active MexT) compared to their parental strain PA (MexS-/MexT-, inactive MexT from XXY genotype) would affect bacterial metabolism. We conducted metabolic phenotyping of these strains. Metabolic phenotyping of 626 compounds using the Omnilog system showed PA∆ and PAn were less metabolically active than PA, particularly in protein, amino acid, and nucleoside metabolism (Dataset S4). These processes are energy-intensive, and their reduction may balance the increased ATP-expensive efflux. Overexpression of *mexEF-oprN* is linked to decreased OprD, which transports basic amino acids and peptides into the cell (Ochs et al. 1999). We observed a 0.8–1.2 log_2_ fold reduction in *oprD* expression in PA∆ and PAn (both MexS-/MexT+) (*P* ≤ .012, Dataset S1), suggesting a genotype–phenotype link, and differences in short peptide utilization. Other systems like the branched chain amino acid transport (*bra*) operon were upregulated in PA∆ and PAn, possibly compensating for reduced *oprD* expression and protein, amino acid, and nucleoside metabolism by utilizing growth medium resources. Differences were not observed in nitrite utilization in PA∆ and PAn despite nitrite gene upregulation.

The shift from central metabolism in antibiotic-resistant strains is logical as efflux is part of the stress response. Without host inducers or antibiotics, quiescent MexEF-OprN may offer fitness benefits. We speculate that nutrient availability in rich media or amino acid-rich environments like the lung (Winstanley et al. 2016), could select for higher metabolic activity in strains with disrupted *mexT* to exploit energy sources. Phosphate availability has been linked to MexT activity, with phosphate supplementation preventing MexT disruption in a rat model (Olivas et al. 2012). Growth of *P. aeruginosa* in peptidic media may require secreted proteases, a quorum-sensing product (Oshri et al. 2018), deficient in efflux-positive strains (Dataset S1). Studies in PAO1 suggest MexT is disrupted to promote RhlR-mediated quorum sensing in LasR-null strains for growth in peptide media (Oshri et al. 2018, Cheng et al. 2022) or to bypass the stringent response and produce virulent strains (Figueroa et al. 2025). We propose *mexS*, not *lasR*, as a driving force. The impact in minimal medium remains untested; further work is needed to determine LasR versus MexS mutations’ impact on efflux shutdown in atypical PAO1. This switch to a more metabolically active phenotype via MexT inactivation is linked to reduced antibiotic resistance but increased virulence.

Overall, metabolic profiling revealed a profound phenotypic switch with active or inactive MexT in PAO1. The impact of *mexS* mutations is unknown. In PAΔ and PAn (both MexS-/MexT+) strains, efflux was switched on by MexT reactivation, similar to others' observations (Juarez et al. 2018). In some strains, the phenotypic switch is governed by MexS inactivation rather than MexT (re)activation (Fig. 1). Future work on our mutant collection’s transcriptomes (Table 2) could unravel the impact of *mexS, mexT*, and *mexEF-oprN* mutations on *P. aeruginosa* physiology.

### Reverting from antibiotic resistance to antibiotic sensitivity is achieved through disruption of MexT

We observed the switch from antibiotic-sensitive to resistant phenotype in PAO1-derived strains [PAΔ, PAn (both MexS-/MexT+)] and passaged clinical isolates on antibiotic media (Table 2). This switch arose from different antibiotic-sensitive genetic backgrounds: nonfunctional MexS/MexT (strains PA, 11) or functional MexS/MexT (strains 10, 20, A) (Fig. 1). We questioned if, without antibiotic pressure, bacteria revert to sensitivity by refunctionalizing MexS or inactivating MexT. We passaged strains selected for chloramphenicol resistance without antibiotic, and sequenced genomes of sensitivity-reverted isolates. Chloramphenicol-sensitive revertants confirmed resistance is unstable during lab passage, with MexT disruption restoring sensitivity (Figs 1 and 3). MexT was inactivated by indels or mutations, sometimes with multiple inactivation strategies in one population (Table 2). In clinical strain 20, an 8-bp insertion caused a frameshift, producing the *mexT* XXY form seen in PAO1, supporting our proposal that PAO1 *mexT* XXY variation results from resistance and sensitivity reversion in lab culture. Revertants were obtained after relatively few passages. In some, *mexT* was undetected, with complete deletion previously identified in lung-isolated strains (Smith et al. 2006, Warren et al. 2011, Hilliam et al. 2017). MexT mutations arose rapidly (within 6 passages) and were dominant, detected by screening fewer than 10 colonies, implying a fitness benefit. MexT disruption to revert to sensitivity was observed in isolates across *P. aeruginosa* phylogenetic tree branches (Fig. 4). SNP analysis assessed genome-wide changes for altered resistance (Dataset S5), but no consistent changes were found, confirming MexT disruption as key. We hypothesized resistant strains revert to sensitivity without antibiotic pressure since downregulation of MexEF-OprN efflux increases OprD porin expression (Fig. 1) for amino acid import. However, an imipenem-resistant strain lacking functional OprD was available (Strain 10, Table 2) and this strain also reverted to susceptibility through MexT inactivation (Fig. 3, Table 2), suggesting a limited role for OprD in the growth conditions used.

To assess if *mexS* and *mexT* mutations affect bacterial fitness, we analysed growth in ancestors, mutants, and revertants of antibiotic-sensitive Strain A and imipenem-resistant Strain 10 (Table 2, Fig. S4). Resistant mutants with *mexS* inactivation outperformed ancestors under antibiotic pressure but underperformed without it (Fig. S4). Revertants’ *mexS/mexT* backgrounds influenced growth: complete *mexS* and *mexT* deletion (Strain 10; revertants A and B) resulted in revertants growing better under antibiotic pressure than ancestors (though not as well as resistant mutants) and outperforming them without (Fig. S4). Conversely, a frame-shifted MexS with a single MexT amino acid change (Strain 10; revertant C) resulted in no difference between the revertant and ancestor under selective pressure but worse growth of the revertant without selection (Fig. S4). Similar results occurred when *mexS* and *mexT* were disrupted by a frameshift, though this revertant (Strain 10; revertant D) was less impaired compared to the ancestor without selection. Finally, the revertant of strain A with frameshift mutations in both MexS and MexT had similar growth to the ancestor with and without selective pressure and achieved the highest cell densities. While specific to our study’s growth conditions, these results indicate that mutation and reversion pathways impact bacterial fitness, suggesting different reversion mechanisms may be selected under varying conditions or genetic backgrounds.

To investigate whether *mexS/mexT*-disrupted genotypes observed *in vitro* were found in clinical strains, we reviewed our clinical collection and identified two genetically similar catheter urine isolates with both *mexS* and *mexT* impaired by frameshift mutations (Strain 4 and Strain 11; Fig. 4, Table 2). These mutations may reflect antibiotic resistance through *mexS* and adaptation to virulence by *mexT* disruption, although mutations during diagnostic processing cannot be ruled out, as changes were observed during laboratory passage. These isolates had high ciprofloxacin resistance (32 µg/ml) due to a T83I mutation in *gyrA* (Bruchmann et al. 2013). The mutation order is unknown, but previous studies have found *gyrA* and *gyrB* mutations more frequently in backgrounds with efflux system mutations than in sensitive wild-type backgrounds (Llanes et al. 2011). If MexT inactivation occurred first, resistance through gyrase mutations upon subsequent antibiotic exposure would produce both virulent and highly resistant strains.

To assess the impact of repeated antibiotic selection reflecting clinical cycles, we used strain 11, which carried the T83I mutation in *gyrA* and nonfunctional MexS and MexT. Using this ciprofloxacin-resistant isolate, we selected for increased chloramphenicol resistance, which arose through a 1-bp deletion causing a frameshift that refunctionalized MexT and increased ciprofloxacin resistance four-fold (from 32 to 128 µg/ml, Table 2). Mutations in gyrases and increased efflux are independent, additive fluoroquinolone resistance mechanisms (Bruchmann et al. 2013) and are associated with the highest resistance. Upon antibiotic-free passage, MexT disruption was observed as a nonsynonymous amino acid change (G233D, Revertant A) or an 8-bp insertion (MexT XXY, Revertant B); however, the strain remained resistant (reverting to its previous ciprofloxacin MIC of 32 µg/ml) due to the gyrase T83I background. The reversible introduction and excision of a short DNA sequence in *mexT* mirrors the PAO1 XY and XXY forms, supporting the proposed PAO1 evolutionary pathway. Once strains acquire *mexS* mutations, switching between antibiotic-resistant, less virulent, and antibiotic-sensitive, more virulent phenotypes occurs via MexT inactivation, not MexS refunctionalization (Fig. 1). Rarely, MexT was disrupted while MexS remained intact (Fig. S2; PAO1 lineages PAO1_VE2, VE13, and H2O); we speculate that MexS reverts to functionality. SNP and SNPEff analyses were conducted on parent and revertant strains to identify whether other genomic mutations influenced the observed effects (Dataset S5). Although no other key changes were noted, further studies are required to understand the broader circumstances leading to the disruption of MexT.

## Conclusions

*Pseudomonas aeruginosa* pathogenesis is often modelled using the reference strain PAO1, which is phenotypically diverse due to variation in *mexT* and differs in *mexS* compared with other lineages. We suggest that antibiotic resistance in PAO1, driven by an ancestral *mexS* mutation, leads to *mexT* plasticity. This evolutionary path was shown *in vitro* with clinical isolates, but its *in vivo* relevance is unknown. In ancestral antibiotic-sensitive *P. aeruginosa* (Fig. 1), resistance arises from MexS inactivation, disrupting efflux control. In resistant strains, virulence and metabolism decrease unless MexT is inactivated, restoring antibiotic sensitivity and increasing metabolism and virulence, but not efflux control, as inactive MexS cannot modulate MexT (Fig. 1, inactivated *mexS/mexT*). Further antibiotic exposure may reactivate MexT, reverting to resistance. This suggests antibiotic cycles drive genetic diversity and adaptation. MexT deletion or genomic region loss is seen in lung-adapted isolates (Smith et al. 2006, Warren et al. 2011, Hilliam et al. 2017) and may result from antibiotic selection. After MexS inactivation, further mutations like in *gyrA* can enhance resistance, allowing efflux downregulation and a switch to a more virulent phenotype while retaining resistance. Although counterintuitive, losing a detoxification system may be of benefit outside of laboratory culture by increasing virulence and nutrient use (Mex-null PAO1) and reducing efflux-related acidification (Olivares et al. 2014), repressing nitrogen respiration (Arat et al. 2015), reducing stress response growth arrest (Tamman et al. 2015), or being compensated by efflux system redundancy. The absence of reversion to an ancestral genotype suggests an evolutionary ratchet driving bacteria between resistant and virulent phenotypes, preventing a return to the ancestral state, at least in laboratory conditions. The reason for this is unclear, but since an antibiotic-sensitive disrupted MexS/MexT strain is more metabolically active than a resistant disrupted MexS strain, it is hypothesized that the ancestral genotype, with active MexS and MexT, has higher metabolic activity and would be outcompeted by a disrupted MexS/MexT strain with inactive pathways, conserving resources. This hypothesis is untested. Active efflux suggests chronic infection as virulence factor production, inducing a host response, is lowered (Lamarche and Déziel 2011, Winstanley et al. 2016) but our evidence also supports *mexT* inactivation as a chronic infection marker (La Rosa et al. 2018). The nature of infection may influence evolutionary trajectory. Initial MexEF-OprD efflux activation may reduce quorum sensing-controlled virulence factor expression, maintained by *lasR* mutations, allowing *mexT* inactivation while keeping low virulence. Clinically, knowing the *mexS/mexT* genotype could guide treatment. If an isolate is antibiotic-sensitive due to active MexS/MexT, using a MexEF-OprD substrate antibiotic could succeed but may select resistant mutants. If sensitivity is due to inactivated MexS/MexT, using such antibiotics might select a resistant virulent strain, harder to eliminate and more harmful. As whole genome sequencing becomes common in diagnostics, using this information will be crucial for targeted treatments, improving outcomes, and preserving antibiotic efficacy.

## Supplementary Material

xtag044_Supplemental_Files

## Data Availability

All Illumina data have been deposited at the European Nucleotide Archive (www.ebi.ac.uk/ena) under project PRJEB18543 (ERP020481) for the transcriptomics data and PRJEB28534 (ERP110744) for the mutant and revertant sequencing data.
