# Supplementary material for: Plasticity in a bacterial global regulatory switch that drives a shift in antibiotic resistance and virulence
Source: FEMS Microbes. 2026 Aug 25;7:xtag044. doi: 10.1093/femsmc/xtag044 (PMC13543918; doi:10.1093/femsmc/xtag044)
Supplement: Supplementary file 1 [file xtag044_supplemental_files.zip › FEMSMC-2025-064.R2 supplementary data - reviews and author response.docx]

**Reviews of manuscript FEMSMC-2025-064**

**Reviewer 1**

**Recommendation: Major Revision**

This manuscript by Correia and colleagues describes how MexS and MexT serve as a switch for antibiotic resistance and virulence functions of P. aeruginosa. The central premise is interesting -- that bacteria are able to turn these functions on or off (through reversible mutations) as environmental circumstances change. The novelty of the observation is buried somewhat by the description of the various PA strains and the RNA-seq.

There are a few concerns to address:

1. a. The RNA-sequencing is insufficiently described. I can't tell, for example, what "DESeq2 output was used for downstream analysis" (page 37, line 39) means. Are the data presented a DESeq2 analysis? If not, what are they? A student's t-test (line 43) is totally inappropriate for RNA-seq and makes no sense with a concomitant DESeq2 pipeline.

b. The choice to use a timepoint of growth for comparison rather than an OD600 is curious, as bacteria may be at different stages of growth at the same timepoint (particularly around the change from exponential to stationary phase).

c. Supplementary data files 1 and 2 are insufficiently annotated for the reader to accurately know what was presented.

2. I found the naming scheme (PA∆, PAn, etc.) to be really hard to follow. If the authors want to use this they should probably repeatedly include the MexT and MexS status in parentheses -- for example, PA∆ (MexS+ / MexT-) or Pan (MexS- / MexT+)

3. The authors refer to publications reporting data after their study was completed (page 11, line 48; page 18, line 32) but those papers were 3 and 5 years ago!

4. Page 16, line 29. There are, in fact, 3 quorum sensing circuits in P. aeruginosa (PMID 40442328 and many others). The authors ignore the quinolone system which is odd since their own RNA-seq (data S1 and S2) support differential regulation of pqsABCDE by MexT variants, which has also been reported by others (PMIDs 19846594 and 41128522)

5. The graphic design of table 2 is poor and should be improved. Among other things, the red/green highlights are not discernible to men with colorblindness.

**Reviewer 2**

**Recommendation: Reject**

The authors describe the relationship between active and inactive states of the gene mexT in Pseudomonas aeruginosa, and resistance to chloramphenicol and ciprofloxacin.

Although the work has some merit, and some aspects of the manuscript are interesting, I find that most of the findings described here are already known, and therefore the work lacks novelty. Some examples of known facts are:

1. Active MexT leads to ciprofloxacin resistance.

2. Inactivating mutations in mexS lead to chloramphenicol resistance.

3. Activating mutations in mexT lead to chloramphenicol resistance.

4. Transcriptional profile of mexT mutants.

5. MexT-active strains are less virulent in vitro and in vivo.

To my knowledge, the only novel part of the manuscript is the metabolic profiling of the MexT strains. However, those results are not described in detail, and this section feels more like a discussion than results. Their evolutionary hypotheses are interesting. However, I do not think they are well-supported. For instance, they describe finding insertions in mexT as the predominant evolutionary pathway to become susceptible to chloramphenicol, however, the sample size might not be enough to support such a statement.

My recommendation to the authors would be to restructure the manuscript to only include the novel sections outlined above; this would improve both the length and the quality of the manuscript.

Other comments:

L36 P8 Please cite the latest WHO report for priority pathogens.

L36 P10 Check again

L15-22 P12 It is well documented that other P. aeruginosa strains, including clinical strains, also commonly have mutations in mexT, having been designated as a mutational hotspot (PMC7618369, PMC7618370).

L27-39 P13 These sentences are misleading. Not because most of the strains had the XY type means that the sequence is conserved. See comment above – mexT is a mutational hotspot.

L31-46 P14 How does finding that activating mutations in mexT in the presence of ciprofloxacin show that inactive MexS XY-MexT is the ancestor to the sensitive PAO1 strains with inactive MexS and MexT?

Figure 4 is mentioned before Figure 3 in the text.

L50-60 P15 Weren’t both variants isolated from in vitro experiments?

L3-5 P16 The antibiotic used in the experiments was chloramphenicol – why would ciprofloxacin resistance be a selective pressure? It might be a secondary effect, but it is not a selective pressure.

The authors should consider shorten the manuscript considerably.

**Associate Editor Recommendation: Major Revision**

Both reviewers identify substantial concerns; however, the issues raised do not rise to the level of rejection.

Reviewer 2 questions novelty, noting that core aspects of MexT activation, mexS inactivation, efflux-mediated resistance, and reduced virulence in MexT-active strains are established in the literature. That assessment is largely accurate. The manuscript’s contribution lies not in redefining MexT biology, but in integrating evolutionary cycling experiments, documenting repeated emergence of XXY insertions during reversion, and framing MexS/MexT as a reversible phenotypic switch in PAO1 and clinical isolates. This evolutionary plasticity angle provides sufficient conceptual value for the journal, provided the claims are moderated and novelty is clearly delineated from prior work.

Reviewer 1 raises important concerns regarding clarity and statistical handling of the RNA-seq data. The description of analytical pipelines (DESeq2, edgeR, t-tests) is currently inconsistent and must be clarified. These issues reflect presentation and methodological transparency rather than clear evidence of flawed data, and appear correctable.

The manuscript requires substantial revision, specifically:

Clearer positioning relative to existing literature

Tempering of evolutionary and ancestral claims

Explicit clarification of RNA-seq statistical methodology

Streamlining and restructuring to focus on the phenotypic switching model

Given that the central data appear technically sound and the manuscript fits the scope of FEMS Microbes, Major Revision is recommended

**EIC Decision: Major Revision**

**Author's Response to Decision Letter for (FEMSMC-2025-064)**

We would like to thank the Editor and reviewers for their comments. We have reproduced these below, and in red have given our responses. Please note that for reviewer 2 it was not always clear which pages they were referring to – we think they may have been using the actual page count as listed in the PDF of the manuscript (i.e. the xth page of that document). We have nevertheless done our best to identify the areas of their concerns and respond accordingly. Major changes to the manuscript made in response to reviewers are shown with track changes or highlighting. The re-wording and re-writing changes that were made to substantially reduce the word count are not shown due to their pervasive nature.

Associate Editor's Comments to Authors:

Associate Editor

Comments to the Author:

Both reviewers identify substantial concerns; however, the issues raised do not rise to the level of rejection.

Reviewer 2 questions novelty, noting that core aspects of MexT activation, mexS inactivation, efflux-mediated resistance, and reduced virulence in MexT-active strains are established in the literature. That assessment is largely accurate. The manuscript’s contribution lies not in redefining MexT biology, but in integrating evolutionary cycling experiments, documenting repeated emergence of XXY insertions during reversion, and framing MexS/MexT as a reversible phenotypic switch in PAO1 and clinical isolates. This evolutionary plasticity angle provides sufficient conceptual value for the journal, provided the claims are moderated and novelty is clearly delineated from prior work.

Reviewer 1 raises important concerns regarding clarity and statistical handling of the RNA-seq data. The description of analytical pipelines (DESeq2, edgeR, t-tests) is currently inconsistent and must be clarified. These issues reflect presentation and methodological transparency rather than clear evidence of flawed data, and appear correctable.

The manuscript requires substantial revision, specifically:

Clearer positioning relative to existing literature

Tempering of evolutionary and ancestral claims

Explicit clarification of RNA-seq statistical methodology

Streamlining and restructuring to focus on the phenotypic switching model

Given that the central data appear technically sound and the manuscript fits the scope of FEMS Microbes, Major Revision is recommended

We would like to thank the Editor for their careful consideration of our manuscript. We have provided detailed responses to the reviewer’s comments below, but in response to the specific requests made above:

We have moved some material from the results section to the introduction to more clearly delineate it as prior knowledge, and have ensured we reference existing literature throughout where relevant to our findings.

We have made edits to clarify that certain evolutionary statements are referring to our experiments rather than to the whole species.

Detailed information on the RNA-seq analyses has been added.

The manuscript has been substantially shortened, particularly in the sections describing the features of the strains and describing RNAseq.

Reviewers' Comments to Authors:

Reviewer: 1

Comments to the Author

This manuscript by Correia and colleagues describes how MexS and MexT serve as a switch for antibiotic resistance and virulence functions of P. aeruginosa. The central premise is interesting -- that bacteria are able to turn these functions on or off (through reversible mutations) as environmental circumstances change. The novelty of the observation is buried somewhat by the description of the various PA strains and the RNA-seq.

Some of the description of the various strains has been moved to the introduction, to highlight previous knowledge. The RNA-seq section has also been shortened, as have the sections describing the strains.

There are a few concerns to address:

1. a. The RNA-sequencing is insufficiently described. I can't tell, for example, what "DESeq2 output was used for downstream analysis" (page 37, line 39) means. Are the data presented a DESeq2 analysis? If not, what are they? A student's t-test (line 43) is totally inappropriate for RNA-seq and makes no sense with a concomitant DESeq2 pipeline.

We apologise for the confusion here and have clarified the RNA sequencing analysis by amending this to the following:

“Transcriptome reads were aligned to the reference genome (NCBI Genbank with accession number NC_002516) by Genomed using TopHat (Kim et al., 2013) and stored in samtools bam file format. Results were provided in FPKM value output. Count data was further analysed using the R program DESeq2 v1.12.3 (Love, Huber and Anders 2014), on R v3.3.1 and edgeR v3.14.0, and genes with log2FC > -1 and < 1 were excluded. Differential expression was assessed using adjusted p-values (p ≤ 0.05, Benjamini-Hochberg corrected).”

b. The choice to use a timepoint of growth for comparison rather than an OD600 is curious, as bacteria may be at different stages of growth at the same timepoint (particularly around the change from exponential to stationary phase).

Thank you for highlighting that we had missed some important information here. We performed growth curves to determine at which time point strains would be in mid-log phase. This turned out to be at 5 hours for all strains. The following text has been added to the start of the section in the methods titled ‘RNA extraction’ to highlight this:

“Initially, growth curves of all strains were conducted to identify the time point at which they would be in the mid-log phase of growth. This was identified to be at 5 hours for all strains.”

We used this approach as opposed to an OD600 approach as some of the strains were also small colony variants (SCVs), for which OD600 may not be a reliable indicator of growth phase due to altered optical properties. OD600 as a normalisation point in this context could introduce bias, as SCVs and wild-type strains may exhibit different OD600 readings despite comparable cell densities. For this reason, we used a time-based protocol, as growth curves confirmed that all strains were at the same stage of growth (mid-log phase) at the selected time point, and to ensure the duration of exposure to the experimental environment across all strains was the same.

c. Supplementary data files 1 and 2 are insufficiently annotated for the reader to accurately know what was presented.

Thank you for noting that better descriptions were required. We have updated the descriptions for Datasets S1 and S2 to give far more information.

2. I found the naming scheme (PA∆, PAn, etc.) to be really hard to follow. If the authors want to use this they should probably repeatedly include the MexT and MexS status in parentheses -- for example, PA∆ (MexS+ / MexT-) or Pan (MexS- / MexT+)

We have updated the text so that in most instances we have now included the MexS/MexT status in parentheses after the strain name, as suggested.

3. The authors refer to publications reporting data after their study was completed (page 11, line 48; page 18, line 32) but those papers were 3 and 5 years ago!

This is correct – the current manuscript is the cumulation of a long period of work in our group. The first mention of this in the manuscript noted above says “after we had performed these experiments”, which remains accurate. The second mention of this previously said “Following our studies” – this has been edited to say “Following conclusion of our data collection” to better indicate this.

4. Page 16, line 29. There are, in fact, 3 quorum sensing circuits in P. aeruginosa (PMID 40442328 and many others). The authors ignore the quinolone system which is odd since their own RNA-seq (data S1 and S2) support differential regulation of pqsABCDE by MexT variants, which has also been reported by others (PMIDs 19846594 and 41128522)

We agree with the reviewer’s comments. This was an oversight on our part. We have corrected the main text as follows:

Regulatory network demonstrates a route to altered virulence

P. aeruginosa has three main quorum-sensing systems, las, rhl, and PQS (Pseudomonas quinolone signal, pqs), important for acute virulence (Shrout et al., 2006; Letizia et al., 2025).

5. The graphic design of table 2 is poor and should be improved. Among other things, the red/green highlights are not discernible to men with colorblindness.

The design of the table has been updated and the green colouring removed to improve accessibility.

Reviewer: 2

Comments to the Author

The authors describe the relationship between active and inactive states of the gene mexT in Pseudomonas aeruginosa, and resistance to chloramphenicol and ciprofloxacin.

Although the work has some merit, and some aspects of the manuscript are interesting, I find that most of the findings described here are already known, and therefore the work lacks novelty. Some examples of known facts are:

1. Active MexT leads to ciprofloxacin resistance.

2. Inactivating mutations in mexS lead to chloramphenicol resistance.

3. Activating mutations in mexT lead to chloramphenicol resistance.

4. Transcriptional profile of mexT mutants.

5. MexT-active strains are less virulent in vitro and in vivo.

To my knowledge, the only novel part of the manuscript is the metabolic profiling of the MexT strains. However, those results are not described in detail, and this section feels more like a discussion than results. Their evolutionary hypotheses are interesting. However, I do not think they are well-supported. For instance, they describe finding insertions in mexT as the predominant evolutionary pathway to become susceptible to chloramphenicol, however, the sample size might not be enough to support such a statement.

My recommendation to the authors would be to restructure the manuscript to only include the novel sections outlined above; this would improve both the length and the quality of the manuscript.

We thank the reviewer for their detail analyses of our manuscript. While we agree that many of these aspects are already known (and we clearly cite these), the direct links between them in defined systems are not. Our paper ties together these aspects of PAO1 biology. We explain the drivers for the inactivation of MexT observed by us and others (i.e. to switch off efflux when mexS is also mutated). Our paper highlights the need to understand (and record) the genetic background of model strains such as PAO1 to fully interpret experimental results. Even recent literature seems to miss some of these unusual aspects of PAO1. With respect to point 3 above, here we refunctionalise MexT, i.e. return it to its intact ancestral form. This is distinct from other ‘activating’ mutations.

To our knowledge, the antibiotic cycling using clinical isolates is also a novel aspect of our work, as is the demonstration that the nature of the PAO1 MexS/MexT mutational profile (and not just their overall functional status) changes laboratory growth characteristics.

Regarding the comment about predominant evolutionary pathways, the only mention of this is where we describe mutations in mexS to become resistant to chloramphenicol. We agree that here, it was not clear that we were specifically referring to within our experiment, and so a statement to this effect has been added. Later in the manuscript where we described changes in mexT resulting in reverting to chloramphenicol sensitivity, statements referring to the fact that we are talking in the context of our experiment are already present.

Whilst there might not be sufficient evidence that insertions in MexT more widely are the predominant evolutionary pathway to chloramphenicol susceptibility (in MexS mutated strains), there is fair evidence in our paper to suggest that inactivation of MexT in general (whether by insertion/truncation/deletion/inactivating mutation) is indeed a dominant mechanism for chloramphenicol susceptibility when MexS is mutated.

We've emphasised the propensity for inactivation of MexT (by any mechanism) when the strain is antibiotic-resistant through MexS mutations. To the best of our knowledge, this rapid change has not previously been demonstrated across non-PAO1 isolates, and is important since the observed changes occur during laboratory passage, potentially have a significant phenotypic impact, and (for PAO1) have been misattributed to co-existing lasR mutations in the past (e.g. Kostylev et al., 2019).

Other comments:

L36 P8 Please cite the latest WHO report for priority pathogens.

We have updated the reference to cite the most recent report.

L36 P10 Check again

Our analyses (and those by others) indicate that P. aeruginosa PAO1 MexS differs from other reference strains and contains an N249D mutation (Supplementary Figure S2, e.g. Lee, Gallagher and Manoil, 2021). Similarly, it has been established that MexT in PAO1 contains an inactivating insertion that differs from most reference strains and is therefore unlikely to represent the ancestral sequence (e.g. Winsor et al., 2016). This does not mean that MexT is not susceptible to mutation/loss during infection, but simply that PAO1 has already undergone laboratory evolution and is no longer a good representative of the ancestral sequence.

We believe the reviewer is specifically referring to the following sentence:

Investigation of the sub-lineages of the common research strain PAO1 revealed mutations in mexS and mexT that are rare in the wider P. aeruginosa population

Edited as follows to better tie in with the previous edit above:

Investigation of the sub-lineages of the common research strain PAO1 revealed mutations in mexS and mexT that differ from the wider P. aeruginosa population

We have also edited the following sentence:

This confirmed that overall mexT was well conserved and that changes from the wild-type XY form of mexT to the mexT XXY genotype exist but are rare, meaning the vast majority of studied strains differ from the PAO1-UW reference strain.

We have edited this to better clarify our message as below:

This supported our proposal that the ancestral form of MexT is XY, meaning the vast majority of studied strains differ from the PAO1-UW reference strain.

L15-22 P12:

It is well documented that other P. aeruginosa strains, including clinical strains, also commonly have mutations in mexT, having been designated as a mutational hotspot (PMC7618369, PMC7618370).

We believe the reviewer is specifically referring to the following sentence:

However, the sequence of the global regulator mexT varies across PAO1 lineages, compared to the wider P. aeruginosa population, with an impact on phenotype

Thank you for this comment.

Here, our focus is the evolution that has occurred in the reference PAO1 which means it no longer reflects the ancestral sequence. Whilst mutations in mexT may arise (for various reasons), PAO1 mexT does indeed differ from most of the other established reference strains and the wider P. aeruginosa population. We believe this is correct and has been demonstrated in our publication and by others (Table 1, Supplementary Table S2) (Klockgether et al., 2010a; LoVullo and Schweizer, 2020).

We agree that MexT has been referred to as a “mutational” hotspot. Our paper explores why mutations in PAO1 MexT occur readily during laboratory passage - we emphasise the role of MexS as a driver. We also refer to changes in MexT in clinical isolates in our paper.

We believe that it’s important to understand the genetic background of reference strains, especially PAO1. In the two papers referenced by the reviewer, it’s unclear which mexT sequence was considered to be "wild-type”. For example, in PMC7618370 variants were called by alignment to PAO1 which contains a frame-shifted mexT sequence (see below, https://pseudomonas.com/feature/show/?id=107762&view=updates). Please also note that we do refer to PMC7618369 in our publication, but propose mexS, rather than lasR or other factors, as a driver based on our findings.

L27-39 P13 These sentences are misleading. Not because most of the strains had the XY type means that the sequence is conserved. See comment above – mexT is a mutational hotspot.

We thank the reviewer for their comment. We have adjusted this part of the manuscript to clarify.

We also note that the ancestral XY form of mexT is supported by evidence in our paper, but also by updates to an established database (pseudomonas.com) where comments were added in 2020 to the PAO1 mexT gene to clarify that an insertion is present (https://pseudomonas.com/feature/show/?id=107762&view=updates) versus other reference strains, leading to a truncated gene product.

We have made the following edit to the text:

Overall 98% of P. aeruginosa were XY for mexT and 91% of mexF sequences lacked disruptive mutations and therefore encoded an inducible efflux system. This confirmed that overall mexT was well conserved and that changes from the supported our proposal that the ancestral form of MexT is wild-type XY form of mexT to the mexT XXY genotype exist but are rare, meaning the vast majority of studied strains differ from the PAO1-UW reference strain.

L31-46 P14 How does finding that activating mutations in mexT in the presence of ciprofloxacin show that inactive MexS XY-MexT is the ancestor to the sensitive PAO1 strains with inactive MexS and MexT?

We would like to thank the reviewer for their comment. We have softened the language here as highlighted below:

Functionalisation MexT in PA∆ and PAn (both MexS-/MexT+) caused resistance to chloramphenicol, ciprofloxacin, and imipenem, consistent with MexEF-OprN pump overexpression without functional MexS (Figure 1, Supplementary Dataset S1 and S2). We propose that antibiotic-resistant PAO1 with inactive MexS but functional MexT (XY genotype) is the likely ancestor of sensitive PAO1 strains with inactive MexS and MexT, where MexT disruptions occurred via various mechanisms (XY* and XXY) (Figure 1, Figure 3).

We have several pieces of evidence to support the proposed evolutionary trajectory.

First, we want to distinguish between “activating” mutations and refunctionalisation of MexT. To the best of our knowledge, there is one key paper (Juarez et al., 2018) that refers to activating mutations in PAO1 MexT. We refer to re-functionalising mutations: these restore the correct reading frame to produce the “wild type”/ancestral MexT sequence and occur when mexT has been disrupted by a short insertion (as observed in PAO1 XXY) and is subsequently exposed to Cm. Importantly, these changes arise in PAO1 with a background mexS mutation, which has been considered the wild type in the past.

An overview of the observed resistance pathways for clinical strains in this experiment is shown in Figure 3. Figure 3 also includes hypothesised changes to PAO1.

These pathways are supported by the following knowledge on PAO1 ancestry. It is known that 1) PAO1 contains a chloramphenicol-resistance mutation in MexS (Lee, Gallagher and Manoil, 2021) and 2) that different lab lineages differ in the integrity of MexT (either active or inactive)(LoVullo and Schweizer, 2020). It is also known that the inactive form of MexT can arise through various mutations, including an 8bp insertion that causes a frame-shift (MexT XXY, Supplementary Table S2). We (and others) have demonstrated that exposure to ciprofloxaxin refunctionalises MexT by reversing the frame-shift. These mutational pathways for PAO1 are summarised in Figure 3.

In our experiments with clinical strains, we saw a similar frame-shift reversal for strain 11 (MexT-inactive, MexS-inactive) upon exposure to Cm.

Figure 4 is mentioned before Figure 3 in the text.

In the version read by reviewers, the first mention in the text of Figure 3 was on page 11, while the first mention of Figure 4 was on page 12. In the revised manuscript, the first mention in the text of Figure 3 is on page 9, with the first mention of Figure 4 on page 10.

L50-60 P15 Weren’t both variants isolated from in vitro experiments?

It is slightly unclear which ‘P15’ the reviewer is referring to – we believe the reviewer may be referring to the 15th page in the PDF document (corresponding to page 12 as numbered on the bottom right of the pages) as this is where their comment would seem to make the most sense. The wording of this section has been modified to clarify that we are referring to the fact that the mexS mutation is seen in the parental, clinical, strain, before the in vitro experiments.

L3-5 P16 The antibiotic used in the experiments was chloramphenicol – why would ciprofloxacin resistance be a selective pressure? It might be a secondary effect, but it is not a selective pressure.

We would like to thank the reviewer for pointing out the confusion here. Their statement is correct, we would not expect to see mutations in topoisomerases or gyrases as the selection was with chloramphenicol. However, as the mutants also had raised ciprofloxacin MICs, we checked for topoisomerase and gyrase mutations. As the manuscript was written, we referred to checking for these mutations before we mentioned that the mutants had raised ciprofloxacin MICs, which is therefore confusing. In order to address this, and the logical flow, we have moved the sentence referring to topoisomerase and gyrase mutations to come after we mention the raised ciprofloxacin MICs, so that it becomes clear as to why we would then check for these mutations.

The authors should consider shorten the manuscript considerably.

We have worked to considerably shorten the manuscript. The main text of the previous version that was reviewed was 9,243 words. This has now been shortened to 7,336 words.

**Reviews of manuscript FEMSMC-2025-064.R1**

**Associate Editor Recommendation: Minor Revision**

Thank you for your thorough revision and detailed response. The reorganization to delineate prior knowledge in the Introduction, the moderation of the evolutionary and ancestral claims, the correction of the quorum-sensing systems, and the added justification for the time-based sampling have addressed the reviewers' main concerns well. I do not feel a further round of external review is needed. However, please address the following minor points:

RNA-seq analysis pipeline. The description in the Methods still does not fully resolve the inconsistency the reviewers and I asked about, and it differs from the wording given in your response. As written, the section states that results were "provided in FPKM value output" and that "count data was further analysed using DESeq2," then that "transcript abundance was quantified using DESeq2 … and edgeR." Please clarify: (a) whether raw counts or FPKM values were the input to DESeq2 (DESeq2 requires raw counts, and the Dataset S1 legend states that count data were provided, which appears to conflict with the FPKM statement); (b) the specific role of each package — which one generated the reported log₂ fold changes and p-values, and what edgeR was used for; and (c) the multiple-testing correction, since the Methods cite Benjamini-Hochberg while the Dataset S1 and S2 legends cite Bonferroni. Please also confirm the role of the Trinity transcriptome assembly step, as it is not obviously connected to the reference-based alignment and counting workflow described.

Consistency of differentially expressed gene counts. The numbers reported for differentially expressed genes are not fully consistent across the manuscript. The Abstract gives ~900 genes; the Results give 600 down- and 327 up-regulated (927 total) "affecting 922/5573," and then refer to 612 and 358 in the overlap analysis; the Dataset S1 legend gives 922 for PA∆ and 815 for PAn, while the Results give 806 for PAn. Please reconcile these figures so that the Abstract, Results, and dataset legends agree.

Superscripts. A number of values appear to have lost their superscripts during file handling — for example, CFU concentrations rendered as "106 CFU/mL" and "5×106 cfu/mL," and the Galleria inoculum given as "ca. 10 total CFU," which cannot be correct as written. Please check the manuscript carefully and confirm that exponents and any other superscript or subscript formatting are intact throughout, and that no numerical errors were introduced, paying particular attention to the inoculum dose in the virulence assay.

**EIC Decision: Minor Revision**

**Author's Response to Decision Letter for (FEMSMC-2025-064)**

Dear Editor,

Thank you very much for your careful consideration of our revised manuscript, and for your comments. We have addressed these as described below:

RNA-seq analysis pipeline:

We would like to thank you for the opportunity to clarify the methods. We have added detail to the methods section to indicate the format that the data was received in, the input that was used for DESeq2, the role of edgeR and Trinity, and corrected the methods to Benjamini-Hochberg where Bonferroni was erroneously stated. We have also ensured this is consistent in the legends.

Consistency of counts:

Thank you for identifying the inconsistencies. We have gone back and recounted, and updated where necessary. The counts are now consistent throughout.

Superscripts:

All instances of missing superscripts or subscripts have been corrected. This includes those pointed out by the editor here as well as others such as in chemical formulae. We can confirm that the Galleria inoculum is ca. 10 total CFU - the Galleria infection model is highly sensitive to P. aeruginosa and so very low infectious doses are required.

**Reviews of manuscript FEMSMC-2025-064.R2**

**Associate Editor Recommendation: Accept**

Thank you for your careful revisions. The manuscript now satisfactorily addresses the concerns raised during review, particularly regarding the RNA-seq analysis and presentation of the study’s contribution, and I do not believe further external review is necessary. One numerical inconsistency should be checked: the Results report 327 upregulated genes in PAΔ but subsequently describe an overlap of 245/358; please verify and correct the applicable count or denominator. Subject to that correction, I support acceptance.

**EIC Decision: Accept**
